# Astrocytes improve tactile categorization by modulating sensory processing in neurons

**DOI:** 10.1016/j.isci.2026.117972

**Published:** 2026-10-29

**Authors:** Alejandra Calero-Vargas, Frida Higinio-Rodríguez, Rafael Olivares-Moreno, Luis Alfredo Rodríguez-Blanco, Victor de Lafuente, Ranier Gutierrez, Gerardo Rojas-Piloni, Oswaldo Pérez, Mónica López-Hidalgo

**Affiliations:** 1 Escuela Nacional de Estudios Superiores, Unidad Juriquilla, Universidad Nacional Autónoma de México, Querétaro, México; 2 Instituto de Neurobiología, Universidad Nacional Autónoma de México, Campus Juriquilla, Querétaro, México; 3 Laboratory Neurobiology of Appetite, Department of Pharmacology, CINVESTAV, Mexico City, Mexico; 4 Laboratory Neurobiology of Appetite, Center for Research on Aging (CIE), CINVESTAV, Mexico City, Mexico

**Keywords:** astrocytes, secondary somatosensory cortex, sensory processing, perception, tactile categorization

## Abstract

Astrocytes are active elements in sensory processing; however, it remains unclear whether they contribute to the perception and categorization of sensory stimuli. Specifically, whether astrocytes in the secondary somatosensory cortex (S2), a higher-order cortical area whose neuronal activity underlies perceptual choices, actively contribute to this process is unknown. Here, we show that chemogenetic modulation of S2 astrocytes improves mice’s performance on a categorization task. Calcium imaging shows that S2 astrocytes respond to vibrotactile stimuli in a nonlinear manner, comprising selective, variable, and inhibited response profiles. Furthermore, chemogenetic modulation of S2 astrocytes bidirectionally adjusts ensembles-specific gain of sensory-evoked neuronal responses, reshapes sensory adaptation and facilitation, and increases the signal-to-noise ratio of sensory-evoked responses. These findings demonstrate that the dynamics and interplay of S2 neuro-astrocyte circuits shape perceptual decision-making during sensory categorization.

## Highlights

• S2 astrocyte modulation improves vibrotactile categorization in mice
• S2 astrocyte calcium responses to vibration are heterogeneous
• Chemogenetic modulation of S2 astrocytes reshapes neuronal ensemble activity
• S2 astrocyte modulation increases signal-to-noise ratio and shapes sensory adaptation

## Graphical abstract

**Figure:**
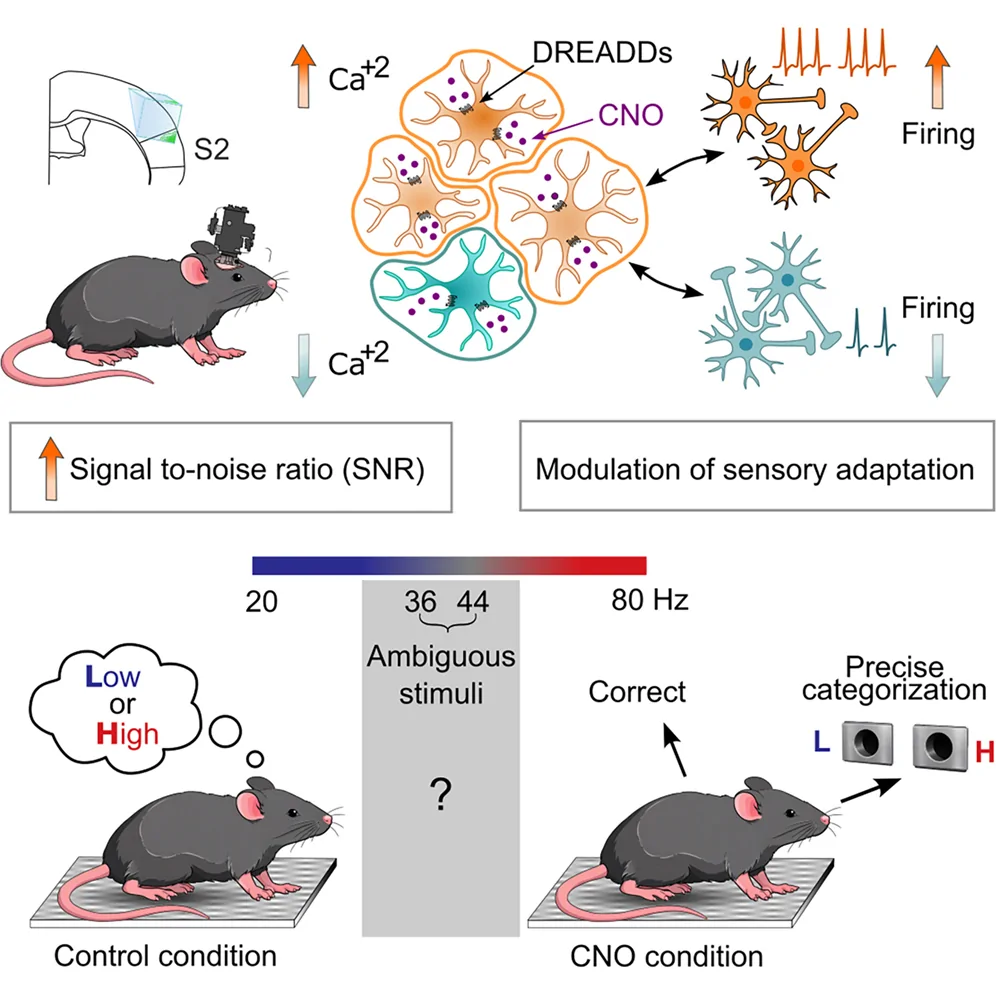

## Teaser

Biological sciences; Neuroscience; Cell biology

## Introduction

Sensory processing enables animals to receive information about the world and guide their behavior. The mechanisms underlying this process have traditionally been thought to rely only on neuronal activity. However, astrocytes play a significant role in sensory processing^1^^,^^2^ and respond to sensory stimuli with intracellular calcium transients across diverse sensory areas.^3^^,^^4^^,^^5^^,^^6^^,^^7^ Astrocytes establish dynamic communication with neuronal circuits and modulate them in turn, at spatial scales ranging from individual synapses to large neuronal populations.^8^^,^^9^ In the somatosensory system, astrocytes located in sensory relays, such as the spinal cord, thalamus, and primary somatosensory cortex (S1), respond to different sensory modalities and encode features of the stimuli, such as modality and intensity.^1^^,^^10^^,^^11^

Tactile information reaches both the primary (S1) and the secondary somatosensory cortex (S2) almost simultaneously.^12^^,^^13^^,^^14^ S2 is a higher-order cortical region that processes tactile information, and encodes perception based in the context of behavior, where sensory and category-related responses co-exist in the same population.^15^^,^^16^ S2 neuronal activity correlates more strongly with perceptual-decision context and behavioral output than with stimulus features. Moreover, S2 neurons integrate sensory information over longer periods, which supports the accumulation of evidence needed to classify tactile stimuli based on differences in intensity, frequency, or location, a process known as categorization.^17^ Although the understanding of astrocyte function in sensory processing is growing, it remains unclear whether they contribute to the perception and categorization of sensory stimuli.

In the present work, we show that S2 astrocytes respond to vibrotactile stimuli in a nonlinear manner, with different response profiles (activated, inhibited, and variable) present across the population. The chemogenetic modulation of S2 astrocytes improves vibrotactile categorization, reshapes the activity of S2 neuronal ensembles, and changes the dynamics of sensory adaptation and facilitation, favoring an increased signal-to-noise ratio of sensory responses. This work sheds light on the active roles of astrocytes in higher-order somatosensory processing that guides perceptual choices.

## Results

### Modulating S2 astrocytes is sufficient to improve vibrotactile categorization

We used a categorization task to evaluate the performance of male mice (C57BL/6, 3 months old) classifying frequencies of vibrotactile stimuli as low or high. First, mice (*n* = 9) were trained over 16 days to discriminate 20 and 80 Hz vibrotactile stimuli (reference frequencies) in a two-port choice by choosing the left or right port, respectively. Once the animals correctly categorized the reference frequencies in at least 80% of all trials (correct trials) (Figure 1A), the categorization was then probed with 6 untested intermediate frequencies (tested frequencies: 24, 30, 36, 44, 54, and 66 Hz) logarithmically spaced between the two reference frequencies accordingly. To get a reward, the mice had to categorize the vibrating stimuli near 20 Hz (24, 30, and 36 Hz) as low by reaching the left port or near 80 Hz (44, 54, and 66 Hz) as high by choosing the right port. Mice achieved their peak performance at 20 and 80 Hz with no significant difference between them (*p* = 0.6), suggesting that mice do not have a preference for distinguishing low and high frequencies within that range. However, when the test frequency was farther from the reference frequencies, as occurred at the midpoint (36 and 44 Hz), the probability of correct trials was lowest (*p* = 0.46) (Figure 1B).

**Figure 1:**
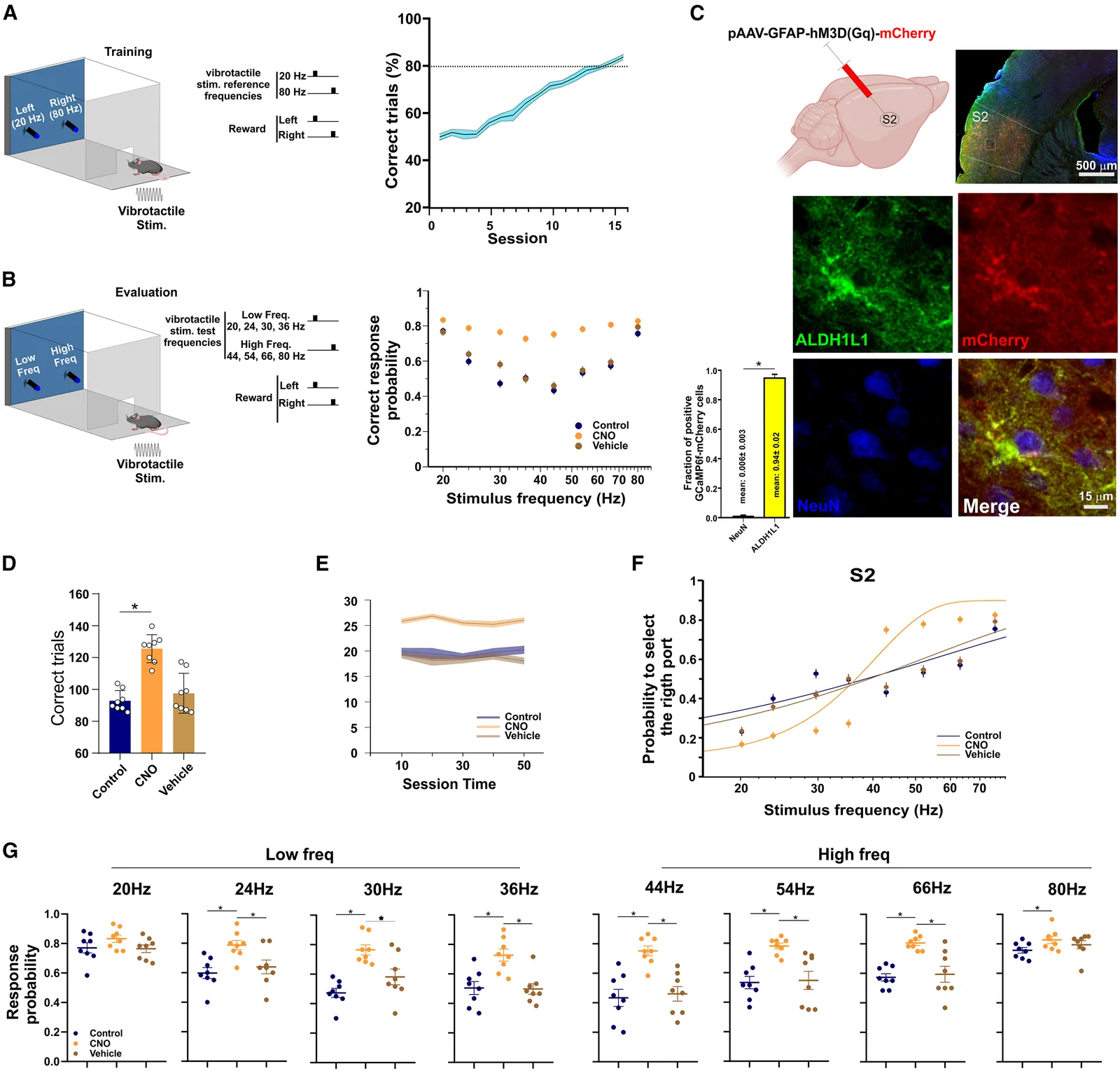
Chemogenetic modulation of S2 astrocytes improves performance in a categorization task (A) Left: behavioral chamber and task sequence. Mice were trained to select the correct port to obtain a reward (left port for 20 Hz vibrotactile stimulus, right port for 80 Hz; *n* = 9 mice). Right: the acquisition curve shows the percentage of correct trials throughout the sessions. The dashed line indicates 80% of correct trials. (B) Categorization task. Left: mice that categorize 20, 24, 30, and 36 Hz as low-frequency stimuli (left port) and 44, 54, 66, and 80 Hz as high-frequency stimuli (right port) receive a reward. Right: probability of correct port selection under control, CNO, and vehicle conditions. (C) Specific expression of DREADDs (hM3D(Gq)) under the GFAP promoter in S2 astrocytes (*n* = 3 mice). Top right: injection site in S2. Immunohistochemistry shows astrocytes (ALDH1L1, green), DREADDs (mCherry, red), neuronal nuclei (NeuN, blue), and merged colocalization. Inset graph indicates the fraction of positive GCaMP6f/mCherry double-positive cells. Scale bars, 15 μm. (D) Total correct trials under control, CNO, and vehicle conditions (∗*p* ≤ 0.05; one-way repeated measures ANOVA). (E) Correct trials throughout the session in 10-min intervals under control, CNO, and vehicle conditions. (F) Psychometric curves showing the probability of correct port selection as a function of stimulus frequency under control, CNO, and vehicle conditions when DREADDs were expressed in S2 astrocytes. (G) Frequency-specific probability of correct port selection throughout the session under control, CNO, and vehicle conditions (one-way repeated-measures ANOVA; ∗*p* ≤ 0.05). Error bars indicate ±SEM.

To investigate the role of S2 astrocytes in the categorization of vibrotactile stimuli, we expressed designer receptors exclusively activated by designer drugs (hM3D(Gq), DREADDs) selectively in astrocytes using the GFAP promoter (Figure 1C). All animals were tested under control, clozapine-N-oxide (CNO), and vehicle (saline) conditions (*n* = 9). DREADDs activation in astrocytes with CNO significantly increased the total number of correct trials, throughout the entire session (control, 92.83 ± 1.64 vs. CNO, 125.6 ± 1.80; *p* < 0.01) compared to the same animals in control or vehicle conditions (Figures 1D and 1E; one-way ANOVA followed by Tukey’s post hoc test, *p* < 0.01). When CNO was administered, peak performance was achieved at the reference frequencies (20 and 80 Hz), with probabilities of correct responses of *p* = 0.83 and *p* = 0.82, respectively. The lowest probability of correct responses was observed at intermediate frequencies (*p* = 0.73), significantly high compared to control and vehicle conditions (Figure 1B; control, *p* = 0.47 vs. CNO, *p* = 0.74 vs. vehicle, *p* = 0.48). This was evident in the psychometric curve, where the probability of selecting the right port was higher (*p* = 0.8) for 44, 54, 66, and 80 Hz and lower (*p* = 0.2) for low frequencies (20, 24, 30, and 36 Hz) (Figure 1F). After CNO administration, but not after vehicle administration, performance in categorizing vibrotactile stimuli significantly increased, except at 20 Hz (control *p* = 0.7 vs. CNO *p* = 0.8) (Figure 1G). To test whether the improvement in categorization was specific to S2 astrocytes or could extend to astrocytes located in other sensory cortical areas, we expressed DREADD in primary visual cortical (V1) astrocytes and the mice’s performance was evaluated under control, CNO, and vehicle conditions. In contrast to S2, CNO modulation of V1 astrocytes did not change the performance at any of the tested frequencies (one-way repeated-measures ANOVA) (Figure S1). Furthermore, CNO administration to wild-type mice without DREADDs expression, did not modify the voluntary locomotor activity in a running wheel compared to pre-injection baselines (Figure S2; unpaired Student’s *t* tests, *n* = 5; per condition *p* > 0.05), excluding nonspecific effects of CNO at the experimental dose. Together, these findings ruled out off-target effects of CNO and are consistent with the regional and circuit-specific role of chemogenetic modulation of S2 astrocytes in mediating the behavioral effect.

### S2 astrocytes integrate vibrotactile information through heterogeneous calcium dynamics

To understand the mechanisms underlying the improvement in the mice’s performance in the vibrotactile categorization task due to chemogenetic modulation of S2 astrocytes, we first analyzed how S2 astrocytes respond to vibrotactile stimuli. We performed chronic calcium imaging of GCaMP6f-expressing astrocytes in anesthetized mice (Figure S3) through microprisms implanted in S2 using a Miniscope (Figures 2A and 2B). Basal calcium activity was imaged for 10 s, followed by 2 s of a vibrotactile stimulus randomly delivered to the contralateral forepaw at the implanted prism (Figure 2C). Sensory-responsive astrocytes were selected based on the amplitude and calcium dynamics under control conditions (see STAR Methods, *n* = 192 astrocytes from 4 animals). At the population level, astrocytes exhibited large trial-by-trial variability (Figure 2D) and responded to all the vibrating stimuli with a mean probability of *p* = 0.33 ± 0.24. In particular, S2 astrocytes showed a higher response probability at both 20 and 80 Hz (*p* = 0.38 ± 0.19 and *p* = 0.39 ± 0.24, respectively), and a significantly lower probability of responding at intermediate frequencies (36, 44, and 54 Hz) (Figure 2E). To further characterize the sensory tuning properties of astrocytes, we analyzed whether astrocytes exhibit frequency-selective responses or have mixed selectivity. We found that 20% of astrocytes were highly selective to vibrotactile stimuli, responding to only one or two tested frequencies. The majority (69%) of astrocytes responded to 3–6 tested frequencies, while 11% were low-selective astrocytes responding to a wide range of frequencies (7–8) (Figure 2F). This heterogeneity suggests that most astrocytes are not limited to encoding stimulus features. Instead, they represent and combine tactile inputs with mixed selectivity, suggesting an integrating role in S2. Next, we assessed whether astrocyte calcium activity shows a linear relationship with stimulus frequency. To do so, we measured the peak time, duration, amplitude, and the area under the curve (AUC) of sensory-evoked calcium activity. None of the variables showed a linear relationship with the frequency of the vibrating stimulus, consistent with the involvement of different peripheral mechanoreceptors that encode distinct frequency ranges of the stimulus (Figures 2G–2J).

**Figure 2:**
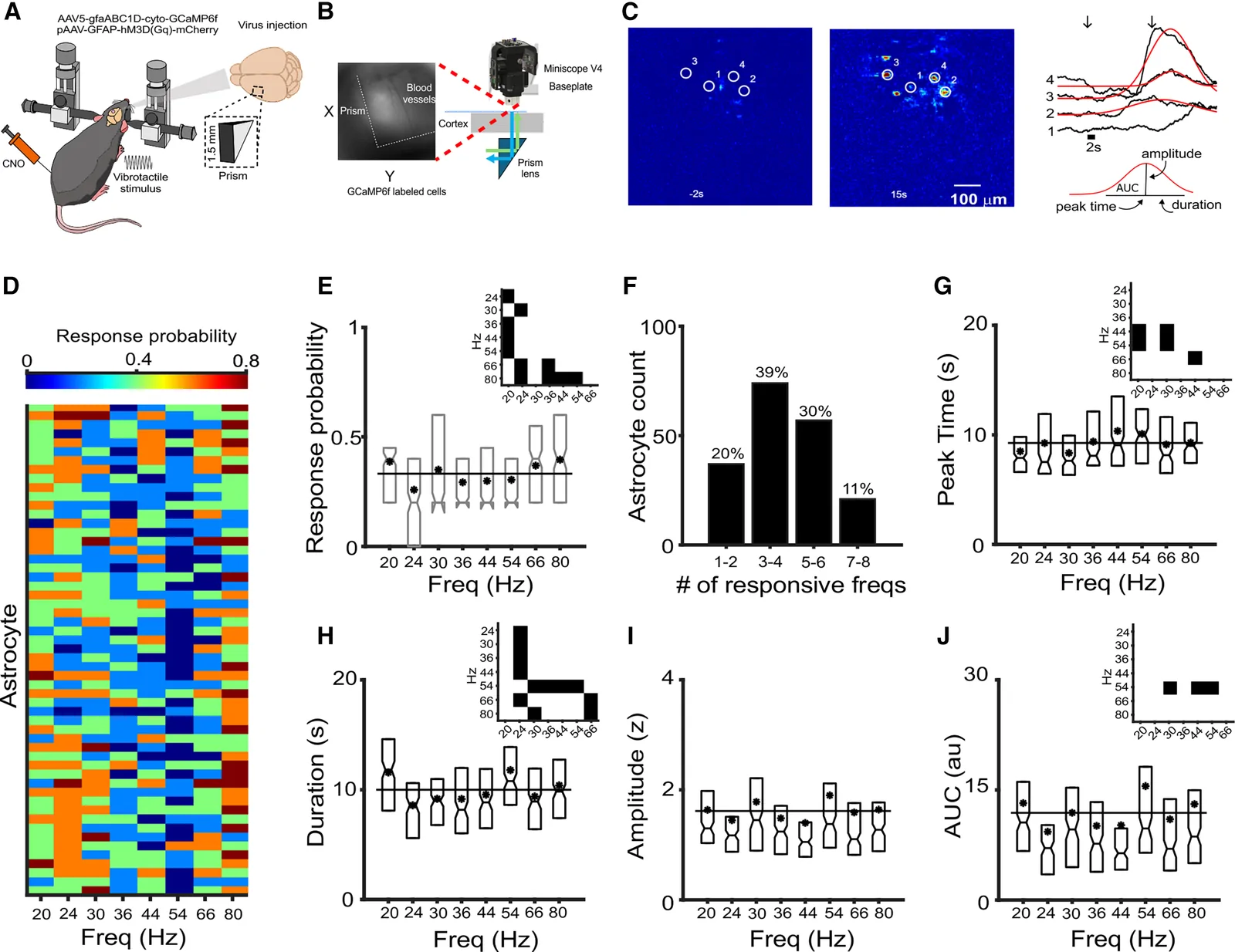
S2 astrocytes are tuned to vibrotactile stimuli through calcium responses (A) Schematic of the experimental design. Vibrotactile stimulation was performed in mice expressing DREADDs in S2 astrocytes before and after CNO injection. (B) Astrocyte calcium activity was imaged using miniscopes coupled with microprisms. (C) Calcium activity in basal and evoked condition. Time (s) indicates time relative to stimulus onset (arrow): 2 s, basal activity before stimulation; 15 s, evoked activity after stimulation. The regions of interest (ROIs) (circles) show four astrocytes and their calcium activity (traces) on the right. Black lines correspond to raw data, and red lines show the Gaussian fit of the responses. The arrows show the moments of image acquisition on the left. The curve (bottom) indicates the parameters derived from the Gaussian fits: amplitude, duration, time to peak, and area under the curve (AUC). Scale bars, 100 μm. (D) Heatmap represents the response probability of astrocytes from one animal as a function of stimulation frequency. (E) Response probability of 192 astrocytes (*n* = 4 mice) to different stimulation frequencies. (F) Histogram of the distribution of the number of frequencies to which the entire astrocyte population responds. (G–J) Mean values per astrocyte of the evoked responses as a function of stimulation frequency: (G), time to peak; (H), duration; (I) amplitude; and (J), AUC. The insets on the top of (E, G, H and J) show post hoc analysis of significant differences between the response probability at different frequencies (one-way ANOVA; *p* ≤ 0.05). In (E and G-J), the dots inside the boxplot indicate the mean value.

To elucidate the mechanism by which CNO improves mice’s categorization of vibrotactile stimuli, we analyzed the effect of CNO on sensory processing in S2 astrocytes. The AUC was computed for each trial using the fitted calcium signal under both, control and CNO conditions. At the population level, chemogenetic modulation of astrocytes by CNO significantly increased sensory-evoked calcium responses by 49% compared to control conditions (AUC: control, 4.01 au vs. CNO, 5.99 au, *p* < 0.001; Figure 3A) without a significant change in their response probability (Figure 3B), suggesting that astrocyte modulation by CNO specifically scale the magnitude of sensory-evoked activity rather than changing the general excitability of astrocytes.

**Figure 3:**
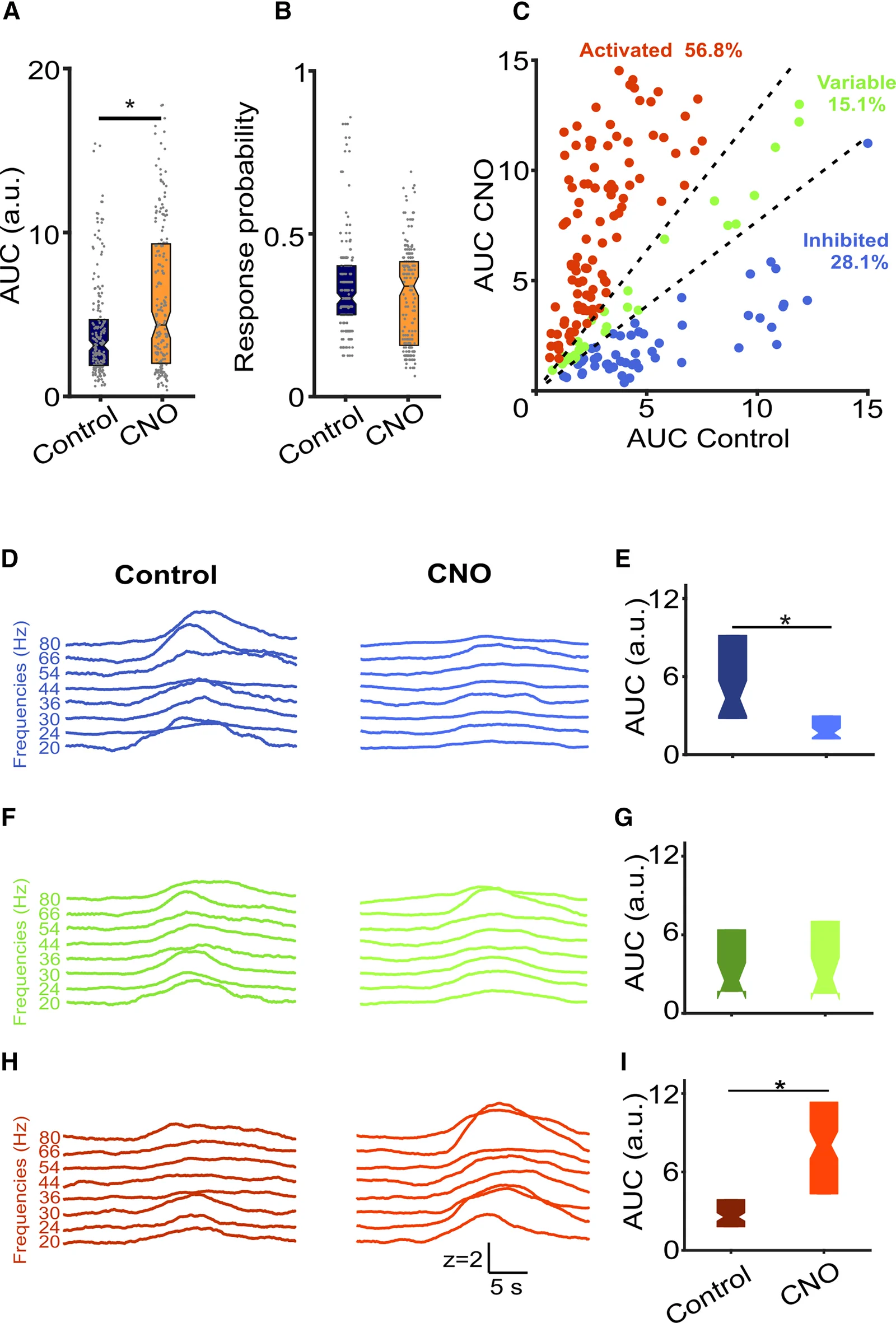
Differential effects of CNO on sensory-evoked calcium activity in S2 astrocytes Boxplots illustrating the distribution of the average area under the curve (AUC) (A) and response probability (B) for all stimulation frequencies in the astrocyte population under control and CNO conditions; each point represents an astrocyte. (C) Scatterplot of the calcium activity (AUC) of individual astrocytes in control and CNO condition, highlighting three distinct subpopulations: activated astrocytes (red), variable astrocytes (green), and inhibited astrocytes (blue). Representative calcium traces of astrocytes for different stimulation frequencies in control (left) and CNO condition (right) for each subpopulation: inhibited astrocytes (D and E), variable astrocytes (F and G), and activated astrocytes (H and I). Boxplots of the mean AUC for each of the three subpopulations defined in (C). *n* = 192 astrocytes from 4 mice. Student’s *t* test; ∗*p* ≤ 0.05.

Beyond the population level enhancement of calcium activity in S2, we analyzed the time-course of the calcium activity of astrocytes induced by CNO as short and long-lasting effects have been reported before (Vaidyanathan et al.).^18^ Astrocytes display heterogeneous profiles. To further elucidate this variability, we classified astrocytes into three groups based on their response to CNO (see STAR Methods) (Figure 3C). The majority of astrocytes were classified as “activated” (56.8%) showing a significant increase in sensory-evoked calcium signal that peak 5 min after CNO administration and returns to baseline activity 30 min after (mean AUC; control, 3.11 au vs. CNO 8.29; Student’s *t* test, *p* < 0.001) (Figures 3H, 3I, and S4E). Another subpopulation, “inhibited” astrocytes (28.1%), reduced their response after CNO administration (AUC: control, 5.70 au vs. CNO 2.33 au; Student’s *t* test *p* < 0.001) (Figures 3D, 3E, and S4C). Finally, the last subpopulation was categorized as “variable” astrocytes (15.1%) because despite displaying a short-lasting increasing in calcium responses (Figure S4D), their overall balanced global session was equivalent between both conditions (AUC control, 4.24 au vs. AUC CNO 4.20 au; Student’s *t* test *p* = 0.96; Figures 3F and 3G).

### Chemogenetic modulation of S2 astrocytes reconfigures population-level frequency selectivity

To explore the functional implications of the heterogeneous sensory-evoked calcium responses, we analyzed the tuning properties of astrocytes within each group. Inhibited astrocytes were divided into eight subgroups based on the mean tuning curve using k-means clustering, with the optimal number of clusters determined by the Calinski-Harabasz index (Figure 4A). All tested frequencies were represented in the entire astrocyte population (Figure 4B), with each astrocyte subpopulation highly selective to one or two frequencies (selective astrocytes). On the other hand, activated astrocytes were classified into two groups with mixed selectivity (Figures 4E and 4F): one group responded under control conditions preferentially to intermediate frequencies and the other to the lowest and highest frequencies. After CNO, both groups showed an overall increase in activity (integrative astrocytes) (Figures 4E and 4F). Finally, variable astrocytes were subdivided into four groups that together covered the full range of frequencies under control conditions with tuning curves (invariant astrocytes) (Figures 4C and 4D). Together, these findings suggest that S2 astrocyte subpopulations have distinct roles in sensory processing, with highly selective astrocytes displaying sensory tuning to stimulus features with precision, and astrocytes with mixed selectivity exhibiting flexible coding, integrating more information that is necessary to adapt their responses to the context.

**Figure 4:**
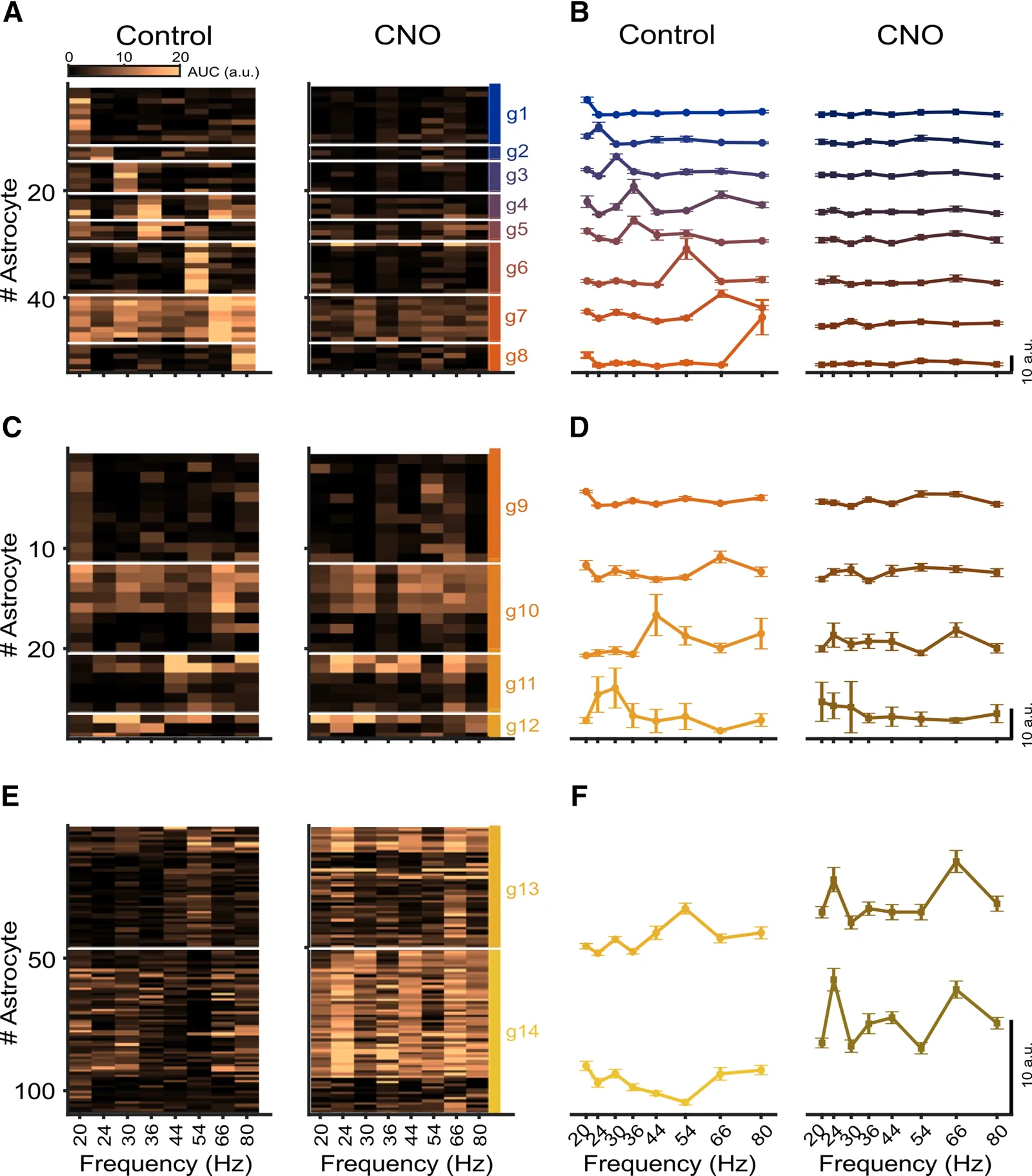
CNO-induced modulation of the tuning curves of astrocyte calcium activity Heatmaps showing the area under the curve (AUC) of calcium responses of individual astrocytes as a function of vibrotactile stimulation frequencies under control (left) and CNO conditions (right). The upper color scale represents the AUC magnitude. Astrocytes are grouped into three subpopulations described in Figure 3, inhibited astrocytes (A); variable astrocytes (C); and activated astrocytes (E). Frequency tuning curves (AUC average), as a function of stimulation frequency for the representative groups of the astrocytes shown in the heatmaps in control and CNO conditions (B, D, and F, respectively). Error bars indicate ±SEM. Groups g1–g8, inhibited astrocytes; g9–g12, variable astrocytes; and g13–g14, activated astrocytes. *n* = 192 astrocytes from 4 mice.

To investigate whether the functional heterogeneity of astrocyte responses is linked to anatomical segregation throughout the column, we examined the spatial distribution of the astrocytes recorded in S2. Although it is not possible to determine the precise location of astrocytes within layers, spatial estimations of the astrocytes within the microprism field of view (FOV) (∼500 μm) indicate that they are distributed on a continuous region of the S2 cortex, revealing an intermingled cellular distribution (Figures S4A and S4B). The mapping of cellular centroids and the analysis of the nearest-neighbor distance reveal that the three subpopulations coexist within the same local microcircuit (Figure S4B). However, the mean distance between activated and variable astrocytes is significantly shorter than that observed for inhibited astrocytes, revealing a non-random spatial organization.

### Astrocyte modulation reshapes ensemble gain and sensory adaptation to improve stimulus encoding

To understand the neuronal mechanism underlying the improvement in vibrotactile categorization due to S2 astrocyte modulation, *in vivo* multichannel electrophysiological recordings were performed in S2 of freely moving mice expressing DREADDs on astrocytes (*n* = 106 neurons from 5 mice) (Figures 5A and 5B). Mice were placed in the behavioral apparatus, where they received vibrotactile stimulation on the paws (Figure 5A). Vibrotactile stimulus frequencies were randomly delivered (20, 36, 44, or 80 Hz), with 15 trials per frequency. These frequencies were selected as the stimuli with the highest (20 and 80 Hz) and lowest (36 and 44 Hz) probabilities of correct choices based on the behavioral data (Figure 1B). We recorded a 10-min baseline period followed by 15 min after saline administration (i.p.) (Figure 5C). After that, 60 trials of vibrotactile stimulation were delivered within 10 min. Each trial consisted of 2 s of vibrotactile stimuli separated by an inter-trial interval of 8 s (10 s total). After completing the saline session, the same protocol was repeated using CNO administration in the same animals.

**Figure 5:**
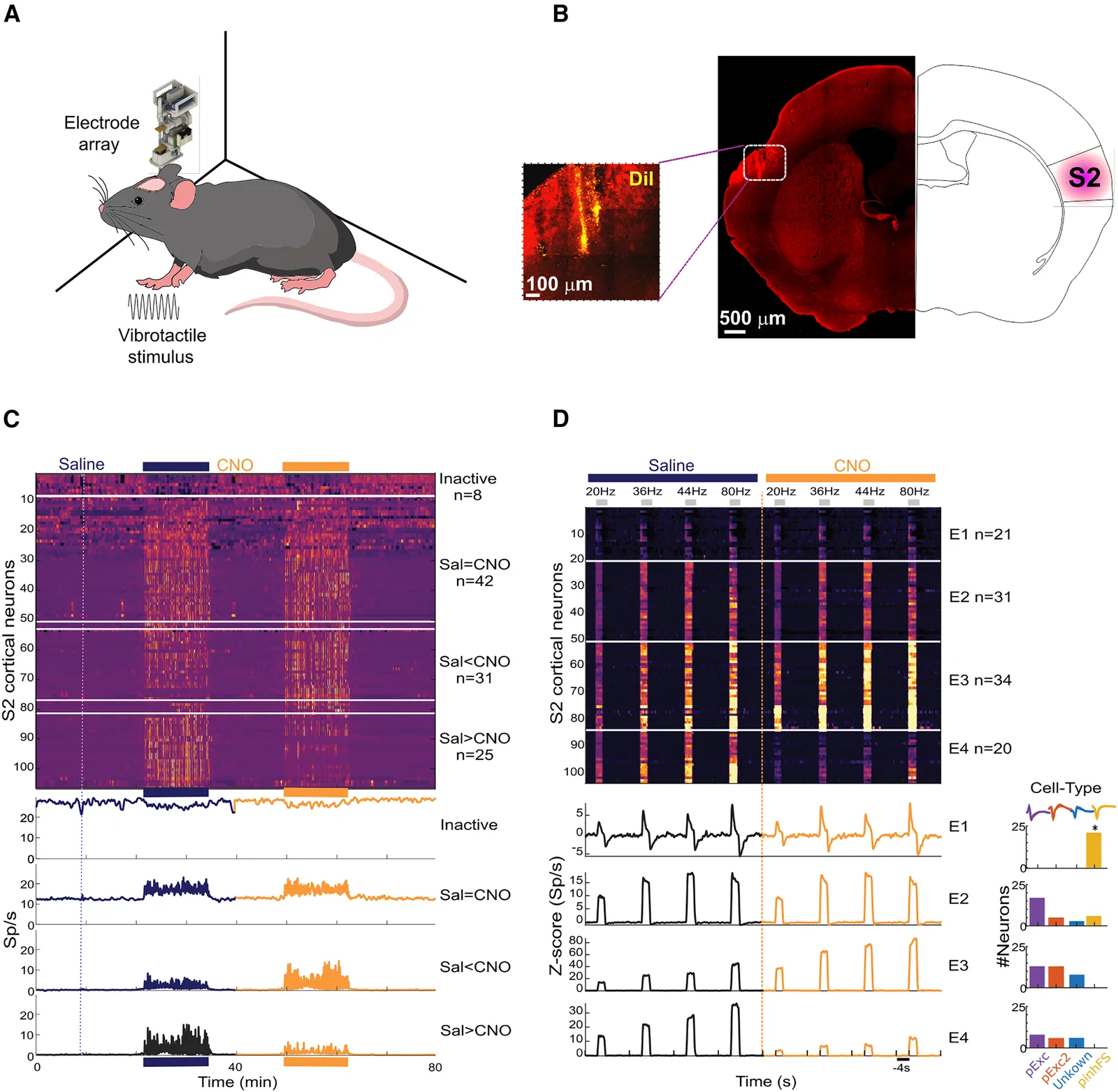
Chemogenetic modulation of astrocytes modulates S2 cortical responses to vibrotactile stimulation in freely moving mice (A) Schematic of the freely moving electrophysiology setup. (B) Multichannel recordings were performed in S2 of five mice expressing DREADDs (hM3D(Gq)) in astrocytes. The inset shows a representative DiI-labeled electrode trajectory. Scale bars, 100 μm (inset) and 500 μm. (C) Upper image: heatmaps (inferno scale: yellow = high activity and purple = low activity) of the neurons recorded (*n* = 106) for ∼80 min per session. After a 10-min baseline, mice received an intraperitoneal injection of saline (blue dashed line), following a 15-min equilibration period, vibrotactile stimulation was delivered (saline condition) where each trial (10 s total), consisted of 2 s of vibrotactile stimulation of randomly selected frequencies (∼20, 36, 44, or 80 Hz) with an 8 s inter-trial interval. After a 15-min interval, the mice received CNO (yellow dashed line), followed by a second stimulation block (10-min). Lower image: t-SNE clustering revealed four neuronal populations, two populations were not modulated by CNO: one was consistently inhibited by sensory stimulation (inactive, putative interneurons; basal firing >30 Hz), and the other robustly activated by the stimulation (Sal = CNO; ∼14 Hz basal activity). The remaining two populations (putative principal neurons; basal firing; <2 Hz) were modulated by CNO, one showed increased sensory-evoked responses under CNO (Sal < CNO) and the other showed decreased sensory-evoked responses under CNO (Sal > CNO). (D) Trial-by-trial analyses of neuronal responses (aligned to each 2 s vibrotactile stimulation) revealed four ensembles. Ensemble 1 (E1) exhibited a sharp peak followed by rapid suppression. Ensemble 2 (E2) was tuned to intermediate frequencies (36–44 Hz). Both E1 and E2 were unaffected by CNO. In contrast, CNO administration amplified ensemble 3 (E3) and suppressed ensemble 4 (E4) responses.

Neuronal activity was analyzed using t-SNE (t-distributed Stochastic Neighbor Embedding), as previously reported.^19^ Four distinct neuronal populations were found based on their responses to vibration and CNO (Figure 5C). The first neuronal population (∼25 *Hz* basal activity) responded to vibrotactile stimulation by decreasing its firing rate and was unaffected by CNO (inactive). A second population (∼14 *Hz* basal activity) displayed robust sensory-evoked responses; the responses were similar between neurons under saline and CNO conditions (Sal = CNO). The last two neuronal populations with a low firing rate, (<2 *Hz*) were modulated by CNO, as they increased (Sal < CNO, third population) or decreased (Sal > CNO, fourth population) their sensory-evoked responses (respectively) to CNO (Figure 5C).

To further dissect these responses, we analyzed the trial-by-trial activity of S2 neurons. To do this, we aligned neuronal responses to the onset of 2-s vibrotactile stimulation. On this rapid timescale, we identified four neuronal ensembles (Figure 5D). Ensemble 1 (E1) exhibited a sharp peak response followed by rapid suppression. A putative classification of cell types based on their waveform characteristics revealed that the E1 ensemble was mainly composed of putative fast-spiking interneurons, suggesting that its activity most likely contributes to the synchronization (gating) of excitatory evoked responses to vibrotactile stimuli. Ensemble 2 (E2), which was tuned to intermediate frequencies (36–44 *Hz*), responded less to lower (20 *Hz*) and higher (80 *Hz*) frequencies. Ensembles 3 and 4 (E3 and E4) responded linearly to the stimulus frequency but differed in their response magnitudes (low and high, respectively). Chemogenetic modulation of S2 astrocytes did not modify the responses of E1 and E2. In contrast, CNO robustly amplified responses in E3 and decreased responses in E4 (Figure 5D). These responses changed linearly with the stimulus frequency. Our results highlight the role of astrocytes as neuromodulators of neuronal circuits, reconfiguring specific ensembles, mainly composed of putative excitatory cortical S2 neurons, in how they process vibrotactile stimuli by tuning the gain of sensory-evoked responses.

One feature in sensory systems is the adjust in the responsiveness of neurons to repetitive sensory stimuli that results in a progressive decrease of sensory-evoked responses (adaptation) to better represent the external world.^20^ We examined whether chemogenetic modulation of S2 astrocytes could affect the adaptation. To this end, we aligned the neuronal responses to vibration onset (time = 0 s), built peri-stimulus firing-rate histograms from −0.5 to 3 s, fitted a least-squares line over the 0–2 s stimulation window and the slope of the fit (m, in Hz·s^−1^) was computed. To quantify this at the population level, we calculate the slope for every neuron during the stimulation-window (*n* = 106) in each condition and tested, neuron by neuron and frequency by frequency, whether the slope changed significantly between saline and CNO. In control conditions, ∼60% of the recorded neurons showed sensory adaptation (negative m), while the rest (∼40%) displayed facilitation (positive m). This is consistent with previous reports in awake animals due to dynamic changes in the behavior and environmental context.^21^

To analyze the role of astrocytes in these processes, we computed the slopes under both saline and CNO conditions for each neuron, using a z-test based on their standard errors and corrected for multiple comparisons across all neurons with the Benjamini-Hochberg false discovery rate (FDR) procedure (*p* < 0.05). Each neuron was classified for each frequency, based on their change in the slope after CNO administration, as decrease (stronger adaptation/weaker facilitation), increase (stronger facilitation/weaker adaptation), or no significant change (Figure 6). Figure 6A shows four representative S2 cortical neurons that illustrate the diversity of CNO effects. The first neuron was essentially unresponsive under saline (80 Hz, m = 0.97) and developed clear stimulus-driven sensory facilitation only after CNO administration, increasingly so at 36, 44, and, most strongly, 80 Hz (positive slope, m = 4.33), indicating that under CNO, this neuron increased its firing rate by ∼4.33 Hz for every second of vibrotactile stimulation. The second neuron displayed sensory adaptation during 80 Hz stimulation in saline (negative slope, m = −1.09), which reverted to facilitation under CNO (m = 5.39). The third neuron exhibited sensory adaptation that remained unaffected by CNO treatment (80 Hz, saline m = −20.8 vs. CNO m = −19.5). Finally, the fourth neuron showed sensory adaptation during 80 Hz stimulation in saline (negative slope, m = −2.1), which was further enhanced under CNO (m = −4.39). Together, these examples reveal that CNO can shift the slope of the stimulus-response function in either direction (adaptation and facilitation) in individual neurons.

**Figure 6:**
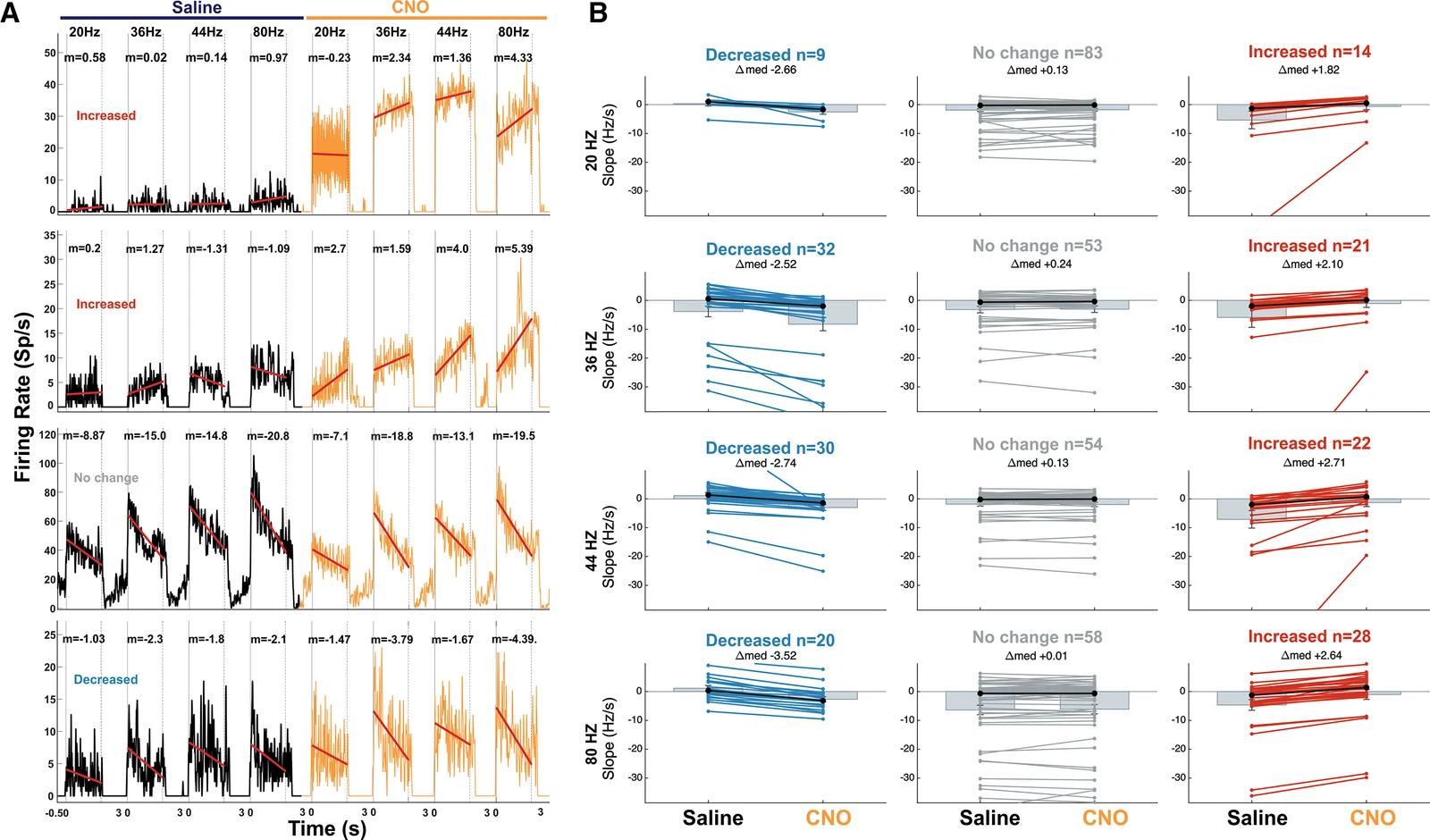
Chemogenetic modulation of S2 astrocytes reshapes sensory adaptation and facilitation (A) Firing-rate responses of four representative S2 individual neurons (rows) to a 2-s vibrotactile stimulus delivered at four frequencies (20, 36, 44, and 80 Hz; columns) under control (saline, black; left) and CNO (orange; right) conditions. Each trace shows the trial-averaged peri-stimulus firing rate (Sp/s), aligned to stimulus onset (time = 0 s); dashed vertical lines indicate stimulus onset (0 s) and offset (2 s). The red line is the least-squares fit over the 2-s stimulation window where m is the slope (Hz·s^−1^). Negative slopes denote sensory adaptation and positive slopes sensory facilitation. (B) Population data of all recorded S2 neurons (*n* = 106) grouped by stimulation frequency (rows) and classified by the statistical change in their slope between saline and CNO into decreased (slope significantly more negative under CNO; blue), No change (not significant; gray) and increased (slope significantly more positive under CNO; red). Thin lines connect the saline and CNO slope of each neuron; bars show the group mean ± SEM, and the thick black line the group median, with the median change (Δmed and Hz·s^−1^) indicated above each image. The number of neurons in each category is given per image (20 Hz: 9/83/14; 36 Hz: 32/53/21; 44 Hz: 30/54/22; 80 Hz: 20/58/28; decreased/no-change/increased, respectively).

At every frequency, a subset of neurons changed their slopes significantly in both directions. The fraction of significantly modulated neurons scale with stimulus frequency: at 20 Hz most neurons were unchanged (decreased/no-change/increased: 9/83/14), whereas at 36, 44, and 80 Hz roughly half of the population changed (32/53/21, 30/54/22, and 20/58/28, respectively). Within the responsive subgroups, the effects were sizable and symmetric in sign: median slope changes (Δmed) in the decreased group ranged from −2.52 to −3.52 Hz s^−1^, and in the increased group from +1.82 to +2.71 Hz s^−1^, while the no-change group remained near zero (Δmed +0.01 to +0.24 Hz s^−1^). Thus, although CNO produced no net shift in adaptation at a population level, S2 astrocyte modulation significantly shape the level of sensory adaptation and facilitation in distinct subsets of S2 cortical neurons.

In order to analyze in more in depth the role of astrocytes regulating sensory adaptation, we used an established paradigm for measuring sensory adaptation in anesthetized animals. We used electrical stimulation (train of 3 electrical pulses of 1-ms delivered at 5 Hz) to provide reproducible stimulus intensities and frequencies, independent of paw contact pressure. This stimulus produces sensory responses very similar to those obtained by mechanical sensory stimulation, with the advantage of delivering reproducible stimuli.^22^ Also, isoflurane anesthesia isolates bottom-up sensory processing by decreasing cortico-cortical top-down modulation that is prominent in awake behaving animals^23^^,^^24^ favoring bottom-up processes. Then, we performed electrophysiological recordings in anesthetized mice (52 neurons from three animals [Figure 7A]. Sensory-evoked responses were calculated as the number of spikes that occurred during 50 ms after the onset of the stimulus [Figures 7B and 7C]). At a population level, repetitive stimulation of the receptive field produces a decrease in sensory responses (sensory adaptation) by ∼30% at the end of the three pulses. DREADDs activation of S2 astrocytes with CNO increased the stimulus-evoked responses while simultaneously decreasing the spontaneous activity (basal activity 200 ms before stimulus onset) (Figure 7D). This dual effect improved the signal-to-noise ratio of sensory responses in S2 neurons (Figure 7E), suggesting that S2 astrocytes fine-tune sensory processing rather than producing generalized effects on neuronal excitability.

**Figure 7:**
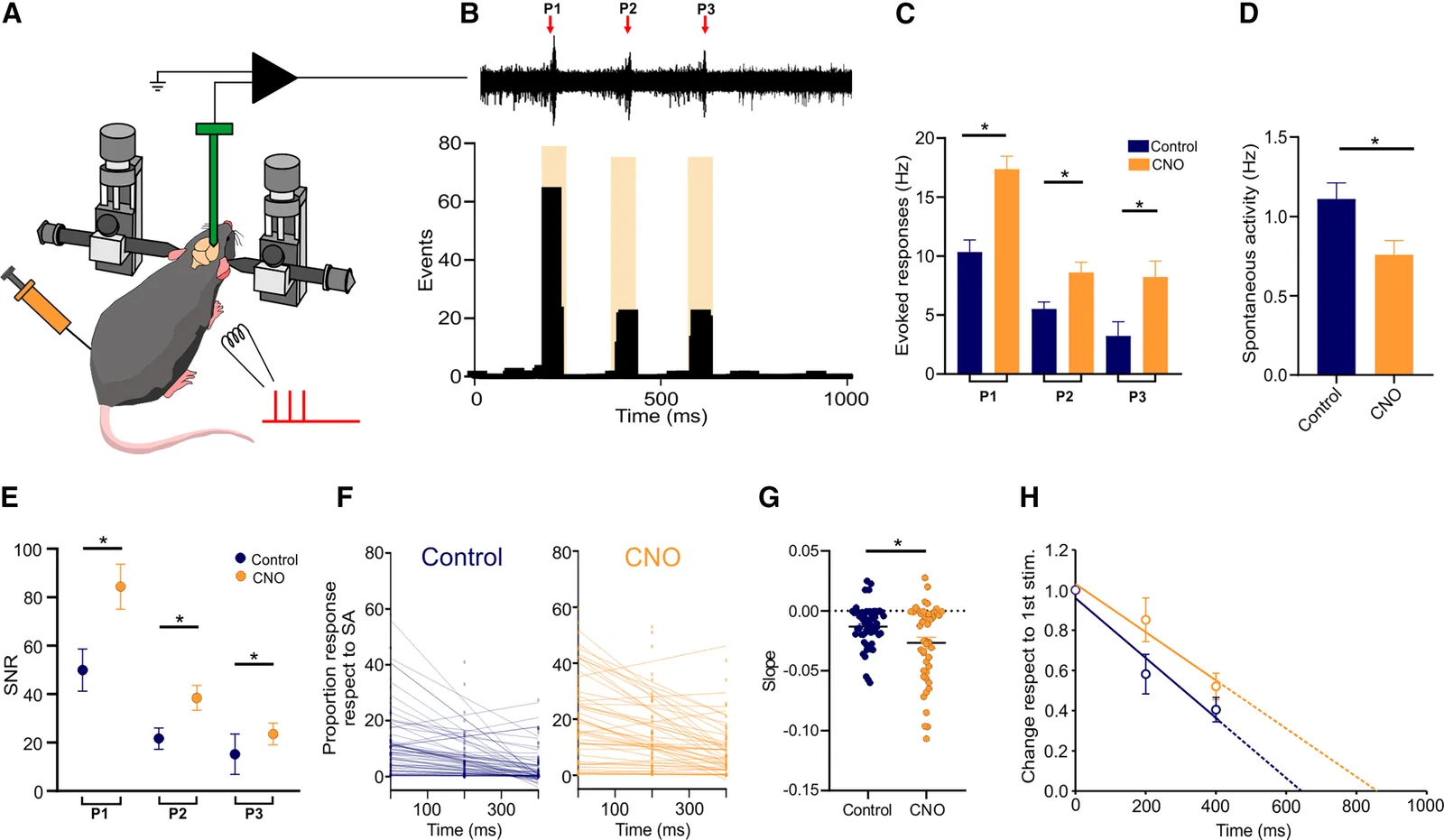
Astrocyte modulation reduces sensory adaptation and increases signal-to-noise ratio in S2 neurons in anesthetized mice (A) Experimental setup showing *in vivo* extracellular recordings in S2 cortical region of anesthetized mice. Sensory-evoked responses were elicited by electrical stimulation of the contralateral forepaw (train of three pulses, 5 Hz). (B) Evoked neuronal activity during the stimulation protocol. Top: representative electrophysiological activity; each stimulus is indicated by red arrows (P1, P2, and P3). Bottom: peristimulus time histograms showing evoked spikes within a 50 ms window (yellow square) after each stimulus. (C) Sensory-evoked firing rates (50 ms post-stimulus) under control (blue) and CNO (yellow) conditions (*p* < 0.05, Mann-Whitney U tests). (D) Spontaneous firing rates (200 ms pre-stimulus) under control and CNO conditions (∗*p* = 0.0291, Mann-Whitney U test). (E) Signal-to-noise ratio (SNR), computed as evoked relative to spontaneous activity, was significantly increased under CNO (∗*p* ≤ 0.05, Mann-Whitney U test). (F) Trial-by-trial proportion of evoked responses relative to spontaneous activity over time (dots). Linear regressions are shown for control (blue) and CNO (yellow). (G) Linear regression slopes comparison derived from normalized evoked responses relative to spontaneous activity (unpaired Student’s *t* test, ∗*p* ≤ 0.05). (H) Normalized changes in evoked responses relative to the first stimulus. Linear regression fits to mean normalized responses are shown, with dotted lines indicating the calculated x-intercept in each condition. Error bars indicate ±SEM. n = 52 neurons from 3 mice.

Among the S2 recorded neurons, the majority displayed sensory adaptation (72.3%), 16.9% of the neurons did not modify their response during the stimulation, while the minority (∼10%) display facilitation. We therefore analyzed the effect of CNO on the slopes of the neuronal activity. Under control conditions, sensory adaptation was evident, with neuronal responses decreasing across repeated stimuli and an estimated cessation of evoked responses at 644.8 ms (time when the x-intercept occurred). However, after CNO administration, a significant reduction in sensory adaptation was observed, and the estimated cessation of sensory-evoked responses was prolonged by 33%, extending to 859 ms (time when the x-intercept occurred) (Figures 7F–7H).

These results suggest that the increase in the performance on a categorization task due to chemogenetic modulation of S2 astrocytes is favored by the increase in the signal-to-noise ratio of sensory-evoked responses. Furthermore, the reduction in sensory adaptation maintained neuronal responses across repeated stimulations, supporting the accumulation of evidence that facilitates the categorization of vibrotactile stimuli. Together, these findings highlight the fine roles of astrocytes in modulating behavioral responses.

## Discussion

Our findings indicate that S2 astrocytes actively modulate higher-order vibrotactile processing, influencing how cortical ensembles engage sensory information during tactile decisions. By combining recordings from awake and anesthetized mice, we identify four mechanisms through which astrocyte activity contributes to tactile categorization. First, chemogenetic modulation of astrocytes in awake animals selectively and bidirectionally reshaped sensory gain within specific ensembles of putative excitatory neurons, while putative fast-spiking inhibitory neurons were largely spared. Second, we show that CNO increased the signal-to-noise ratio and decreased sensory adaptation in anesthetized mice, facilitating the accumulation of sensory evidence. Third, calcium imaging revealed heterogeneous responses of the S2 astrocyte population to CNO administration with opposing trajectories of sensory-evoked responses. Fourth, chemogenetic modulation of S2 astrocytes improved mice’s performance in categorizing vibrotactile stimuli, particularly at intermediate frequencies, where mice’s performance was lowest. We propose that S2 astrocytes operate as a state-dependent gain-control system that refines cortical sensory representations to facilitate perceptual decisions.

### Heterogeneous vibrotactile-evoked calcium responses in S2 astrocytes are consistent with a sensory and internal readout

S2 astrocytes display heterogeneous responses to different vibrotactile frequencies. While some S2 astrocytes show high selectivity, responding robustly to only one or two specific vibrotactile frequencies, others exhibit mixed selectivity, responding to a broader range of frequencies. This heterogeneity indicates that S2 astrocytes do not rely on a single coding scheme, but operate as a population in which high- and low-selectivity tuning curves coexist. The low response probability (∼0.3) and high variability observed in our astrocytic calcium signals are inconsistent with direct sensory encoding pathways. Instead, this distinct functional heterogeneity aligns with a highly localized modulatory role. In parallel, CNO administration reveals a second, functionally distinct axis of variability classifying astrocytes into activated, inhibited, and variable response roles. In the peripheral somatosensory system, mechanoreceptors are specialized across distinct frequency ranges. Rapidly adapting Meissner corpuscles and Pacinian corpuscles are sensitive to frequencies in the 20–400 Hz range, with Meissner corpuscles responding to lower frequencies and Pacinian corpuscles to higher frequencies.^25^^,^^26^ The convergence of S2 astrocyte responses to both low and high frequencies regions of the spectrum, suggest that astrocytes integrate diverse somatosensory inputs in higher-order processing.

Our findings position vibrotactile discrimination in S2 as a balance between the sensory readout and the internal readout system. We propose that highly selective and invariant populations of astrocytes act as the main sources of sensory readout. In the control condition, the high tuning of the selective population generates a continuous map of the vibrotactile frequency spectrum. The CNO-induced reduction of selective activity in this population reveals that this tuning depends on a basal activity level that is reduced by CNO, weakening the sensory readout and bringing into focus the contribution of invariant astrocytes. The invariant population spans the full spectrum of vibrotactile frequencies in both control and chemogenetic conditions, with its broader variability in selectivity establishing a baseline modulated by the internal system, represented by the integrator population, which gains predominance under these conditions (Figure 8).

**Figure 8:**
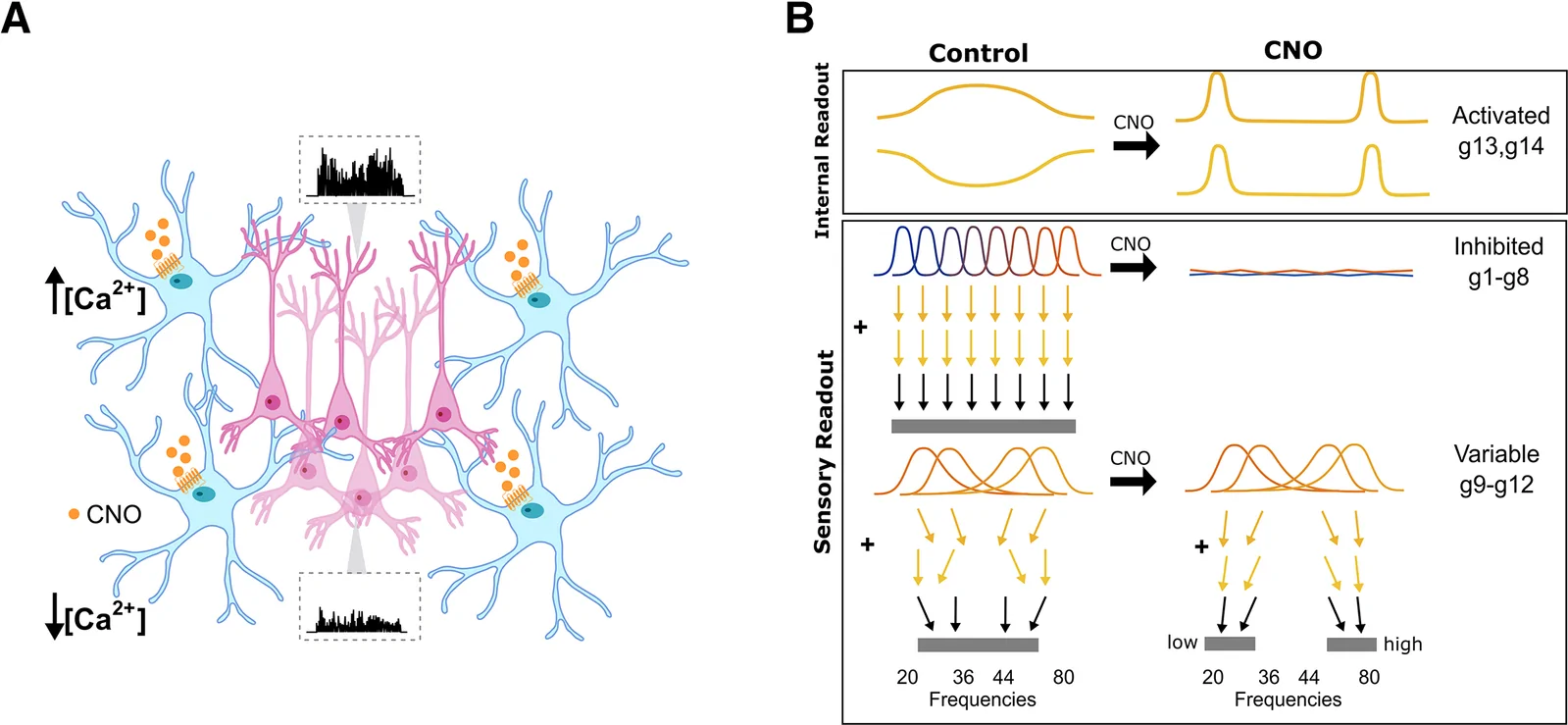
Conceptual framework of CNO-induced neuron and astrocyte network dynamics in S2 facilitating vibrotactile categorization (A) CNO administration induces heterogeneous non-linear sensory-evoked calcium responses across the S2 astrocyte population engaging distinct subpopulations in opposing directions. In parallel, distinct functional ensembles of putative excitatory cortical neurons respond to chemogenetic activation of S2 astrocytes with linear modulation that adjusts the gain of sensory-evoked responses, either enhancing or decreasing their sensory-evoked activity. (B) Schematic representation of the tuning curves of S2 astrocyte calcium responses within the population in control conditions and after CNO administration. Upper image: we hypothesized that the activated astrocytes subpopulation serves as an internal readout mechanism. Under control conditions, this population bifurcates into two distinct groups tuned to intermediate frequencies and to spectral extremes, respectively. However, under CNO, a potentiation of these groups induces a convergence toward the 24 and 66 Hz limits. Lower image: the sensory readout is depicted as being mediated by the inhibited and variable astrocyte subpopulations. Whereas fine-tuned sensory representations are observed under control conditions, our findings are consistent with CNO administration diminishing this frequency resolution. In our model, the influence of the activated units could redirect the activity of “variable” astrocytes toward the internal readout peaks, thereby transforming the sensory map into a bimodal representation that may support enhanced performance in categorization tasks.

Under control conditions, the integrator population splits into two clusters: one tuned to intermediate frequencies and the other to the spectrum extremes. Under chemogenetic modulation of astrocytes, these clusters amplify and align their response to 24 and 66 Hz, forming a perceptual reference point. Within this conceptual model, the sensory encoding of invariant astrocytes is redirected toward the internal system’s salient peaks. The shift from a uniform continuum under control conditions to a bimodal pattern under chemogenetic modulation of astrocytes, could therefore account for the improvement observed in the categorization task, in which boundary delineation supersedes detailed intermediate resolution (Figure 8). However, given the heterogeneous responses of astrocyte subpopulations to CNO, the present data do not allow us to determine which astrocyte population is functionally relevant for the observed neuronal effects. Future studies combining subpopulation-specific manipulation of astrocyte calcium activity with simultaneous neuronal recordings will be necessary to establish the causal contribution of each group to tactile categorization.

The responsiveness of astrocytes to sensory stimuli extends beyond sensory input. Astrocytes are wired to process multimodal information, from local inputs, top-down and bottom-up information, to neuromodulator signals.^23^^,^^27^^,^^28^^,^^29^ In this context, astrocytes express membrane receptors for all neuromodulators and can amplify neuromodulatory effects on neuronal networks by modulating their activity.^30^ Particularly, astrocytes respond to noradrenaline released by the locus coeruleus due to the expression of noradrenaline receptors, leading to an increase in calcium activity.^31^^,^^32^^,^^33^ These factors processed within astrocytes introduce variability and non-linearity into calcium responses, as evidenced by the trial-by-trial variability in S2 astrocytes. Beyond relaying sensory inputs, this neuromodulatory context suggests that astrocytes sit at the interface between bottom-up sensory signaling and top-down state-dependent modulation, providing a candidate substrate by which arousal or task-related signals could shape cortical gain during categorization. Our chemogenetic activation likely recruits, at least in part, intracellular cascades shared with those coupled to endogenous neuromodulator receptors, which could contribute to the bidirectional modulation of excitatory ensembles observed in awake recordings. Finally, an important methodological consideration is that our calcium imaging was performed under light isoflurane anesthesia, a condition that decreases the overall astrocyte calcium excitability while preserving sensory-evoked activity.^34^^,^^35^^,^^36^ Also, locomotion-driven astrocyte recruitment has been reported in other sensory areas^37^^,^^38^ and could not be assessed under these conditions, which is a limitation in the extrapolation of our findings to the awake state. In this context, it would be important to analyze astrocyte calcium dynamics in freely behaving animals.

Our estimates based on the microprism FOV indicate that the imaged S2 astrocyte subpopulations (activated, variable, and inhibited) reside within a continuous columnar region of the S2 cortex, intermingled rather than segregated, with inhibited astrocytes seemingly deeper that activated and variable ones. The present data resolve only depth compartments, and do not allow precise assignment to specific layers. This depth pattern nonetheless suggests that layer-specific astrocytes may exist within S2 and a detailed analysis of layer-circuits within S2 columns will be an important next step.

### Astrocytes fine-tune vibrotactile responses through cell-type-specific gain modulation

Astrocytes modulate sensory-driven behaviors in primary cortical areas.^1^^,^^36^^,^^39^ In S1, astrocytes modulate the magnitude of sensory-evoked neuronal activity and optimize the processing of sensory information, adjusting the dynamic range of cortical responses to sensory inputs.^1^^,^^36^ In the barrel cortex, astrocytes modulate sensory processing.^39^ Tactile discrimination of whisker stimulation is affected when astrocyte N-methyl-D-aspartate receptors (NMDARs) are reduced. This is associated with increased spontaneous neuronal activity, reduced neuronal recruitment, and sensory adaptation. In the present work, the improvement in vibrotactile discrimination produced by chemogenetic modulation of S2 astrocytes depends on the increase in signal-to-noise ratio, a fine mechanism to change the gain of sensory-evoked responses. *In vivo* electrophysiology shows that this modulation is not uniform across neuronal ensembles where some appear amplified, others suppressed and others seemingly unaffected.

Previous studies have shown that astrocytes regulate excitatory and inhibitory neurons in different ways, rather than applying a uniform change in gain across cell types. In the thalamus, astrocytic GABA release acts as a circuit-specific mechanism that adjusts the gain of thalamocortical relay neurons without globally silencing the local network, an effect restricted to inhibitory microcircuits that is not observed in excitatory populations.^11^ In cortex, astrocytes adjust the dynamic range of activity by selectively modulating inhibitory transmission as a function of cortical layer and stimulus modality.^1^ Our waveform-based putative classification of S2 ensembles extends this principle to higher-order sensory cortex. Here, chemogenetic activation of S2 astrocytes predominantly engages putative cortical excitatory microcircuits while putative fast-spiking inhibitory ensembles appear largely spared, supporting a unifying view in which astrocyte-to-neuron signaling is tuned to the specific microcircuit in which it operates.

Sensory adaptation prevents saturation of the neuronal responses to familiar stimuli.^11^ However, this process can limit the encoding of information over time. This principle extends beyond cortical circuits. In the olfactory bulb, astrocytes mediate sensory processing through the transcription factor Sox9, which regulates glutamate clearance via the astrocyte-specific transporter GLT-1. Its conditional deletion dissociates the detection from discrimination. Mice lacking Sox9 displayed higher odor sensitivity and required 100-fold lower concentrations to detect (R)-limonene. However, in parallel, mice showed impaired discrimination of chemically similar odorants.^40^ Together, these findings position astrocytes as intermediates between sensation (detection) and perception (discrimination), suggesting that astrocytic modulation of sensory processing preserves coarse sensitivity at the expense of fine perceptual resolution when astrocyte function is compromised.

In our hands, S2 astrocytes shaped sensory processing through complementary, state-dependent mechanisms. In anesthetized mice, astrocyte modulation reduced neuronal sensory adaptation, extending the temporal window during which sensory information could be processed. In contrast, in freely moving animals, the same manipulation produced heterogeneous, bidirectional modulation of sensory-evoked responses, with some ensembles showing amplified slopes and others showing reduced slopes. This state-dependent bidirectional modulation suggests that top-down inputs and neuromodulatory systems re-engage distinct cortical pathways during wakefulness, recruiting astrocyte function in a more state-dependent manner than during anesthesia.^11^^,^^24^ This pattern positions S2 astrocytes as flexible and dynamic elements. Future studies combining simultaneous calcium imaging and electrophysiology in awake animals may further clarify the spatiotemporal mechanisms by which S2 astrocytes exert these modulatory effects during naturalistic behavior.

### Astrocytes improve vibrotactile categorization

The role of astrocytes in modulating sensory-driven behaviors has been shown in primary sensory cortices.^1^^,^^36^^,^^39^ Beyond primary sensory areas, higher-order processing areas, such as S2 integrate somatosensory information over extended periods, which is important for the categorization of tactile stimuli along dimensions, such as intensity, frequency, or location.^17^ Indeed, S2 neuronal activity correlates strongly with perceptual decisions and the behavioral readout that guides perceptual choices.^14^^,^^16^^,^^41^ Consistent with this framework, our behavioral results show that chemogenetic modulation of S2 astrocytes with CNO improved the mice’s performance in categorizing vibrotactile stimuli into low or high frequency classes throughout the session, particularly at intermediate frequencies where mice perform is lowest, with minimal effect at reference frequencies (20 and 80 Hz). We interpret this pattern through the lens of Weber-Fechner scaling, in which vibrotactile perception follows a logarithmic relationship with stimulus magnitude, placing the highest cognitive demand at intermediate frequencies leaving reference stimuli at the saturated endpoints of the psychometric function. Accordingly, S2 astrocytes appear to operate as a state-dependent gain control mechanism, optimizing perceptual performance precisely where evidence accumulation is most needed, without perturbing already saturated endpoints of the psychometric functions. This effect is region- and circuit-specific to S2, as CNO modulation of V1 astrocytes did not alter performance in the categorization task and wild-type locomotor controls showed no off-target CNO effects, arguing that the behavioral gain reflects the engagement of the S2 neuro-astrocyte circuit rather than general changes in arousal, locomotion or sensorimotor performance. Our findings therefore suggest that the neuro-astrocyte circuit in S2 is a key determinant of perceptual decision-making in sensory-driven tasks, operating by coupling astrocyte-dependent modulation of signal-to-noise ratio and cell-type-specific (excitatory/inhibitory) microcircuits to the temporal accumulation of sensory evidence required for tactile categorization.

The improvement in the performance during vibration discrimination and categorization could potentially be achieved by engaging S2 astrocytes under physiological conditions, for example, during attentive states, in which astrocyte activity acts as mediators of arousal-signaling.^42^^,^^43^ Rather than operating at maximum capacity, the brain appears to recruit astrocyte activity to make fine state-dependent adjustments to behavior based on current needs, optimizing information processing and decision-making without saturating the perceptual readout.

Together, our findings support a critical role for S2 astrocytes in higher-order sensory processing by dynamically shaping signal-to-noise ratio, ensemble-specific excitability, and sensory adaptation to enhance vibrotactile representations and improve perceptual outcomes.

### Limitations of the study

We acknowledge several caveats in the present study. First, only male mice were used in this study, so possible sex differences could not be assessed, studies including both sexes will be needed to determine whether these findings generalize. Second, we did not directly compare CNO-treated control mice (without DREADD expression) with CNO-treated DREADD-expressing mice; CNO in DREADD-negative mice was tested only in locomotion, and the V1/CNO control covered only the categorization task. Third, given the heterogeneous responses of astrocyte subpopulations to CNO, our data do not allow us to determine which astrocyte population is functionally relevant for the observed neuronal effects. Fourth, astrocyte calcium imaging was performed in anesthetized animals, which may not fully capture astrocyte dynamics during awake behavior. Future studies combining subpopulation-specific manipulation of astrocyte activity with simultaneous neuronal recordings and including CNO-treated DREADD-negative cohorts across all experimental paradigms, will be required to address these points.

## Resource availability

### Lead contact

Requests for further information and resources should be directed to and will be fulfilled by the lead contact, Mnica López-Hidalgo (lopezhidalgo@unam.mx).

### Materials availability

This study did not generate any unique reagents.

### Data and code availability

• All data reported in this paper will be shared by the lead contact upon request
• This paper does not report original code.
• Any additional information required to reanalyze the data reported in this paper is available from the lead contact upon request.

## Acknowledgments

A.C.-V. and F.H.-R. are doctoral students of the Programa de Doctorado en Ciencias Biomédicas at Universidad Nacional Autónoma de México (UNAM) and received fellowships (no. CVU 786125 and 1086257) from Secretaría de Ciencias, Humanidades, Tecnología e Innovación (SECIHTI, formerly CONAHCYT). We thank Jessica Gonzalez Norris for proofreading the manuscript and Cutberto Dorado, Nydia Hernández, Ericka de los Rios, Alejandra Castilla, Martín Garcia Servin, Christian Josué Delgado Guzmán, Nuri Aranda, and Moises Mendoza for providing technical assistance. This work was supported by grants UNAM-PAPIIT IN201624 (G.R.-P.) and UNAM-PAPIIT IN217225 (M.L.-H.).

## Author contributions

A.C.-V., F.H.-R., R.O.-M., and L.A.R.-B. performed the experiments, analyzed and interpreted the data, and contributed to the design of the study. V.d.L. contributed to the conceptualization and analysis of the behavioral data. G.R.-P. and R.G. contributed to the design of the study, the analysis of the electrophysiological results, and the interpretation of the data. O.P. analyzed the calcium imaging data, contributed to the design of the study, and participated in the interpretation and integration of the data throughout the paper. M.L.-H. acquired funding, contributed to the design of the study, supervised and led the project, and participated in the interpretation and integration of the data throughout the paper. All authors wrote and reviewed the manuscript and the figures and contributed to the discussion of the data.

## Declaration of interests

The authors declare no competing interests.

## STAR★Methods

### Key resources table

**Table:** 

REAGENT or RESOURCE	SOURCE	IDENTIFIER
**Antibodies**		

Mouse anti-NeuN	Millipore	Cat# MAB377; RRID:AB_2298772
rabbit anti-ALDH1L1	Abcam	Cat# ab177463; RRID:AB_2811300
goat anti-Rabbit Alexa 488	Thermo fisher scientific	Cat# A11008; RRID:AB_2576217
goat anti-Mouse Alexa 647	Thermo fisher scientific	Cat# A21235; RRID:AB_2535804

**Bacterial and virus strains**		

AAV5-gfaABC1D-cyto-GCaMP6f	Addgene	Cat# 52925
pAAV-GFAP-hM3D(Gq)-mCherry	Addgene	Cat# 50478

**Chemicals, peptides, and recombinant proteins**		

SlowFade Gold Antifade Mountant	Thermo Fisher Scientific	Cat# S36937
Clozapine N-oxide (CNO)	APExBIO	Cat# B8466
Triton^TM^ X-100	Sigma Aldrich	X100

**Experimental models: Organisms/strains**		

Mouse: C57BL/6	Animal facility of the Instituto de Neurobiología, UNAM	NA

**Software and algorithms**		

MATLAB version R2025B	MathWorks	https://www.mathworks.com/products/matlab.html RRID:SCR_001622
OpenBridge software	Tucker-Davis Technologies	NA
Offline Sorter	Plexon, TX	NA
Open Ephys Software	Open Ephys	https://open-ephys.org/

**Other**		

Miniscope V4	UCLA	NA
microprism 1.5 mm	Tower Optics	NA
optodrive array	Caballero-Ruiz et al.^44^	
64-channel PZ5 Neurodigitizer amplifier	Tucker-Davis Technologies	NA
RZ2 BioAmp Processor	Tucker-Davis Technologies	
silicon-based multielectrode probe	Neuronexus Technologies	A4x8-5mm-50–200–177-A32 configuration
Master 8 stimulator	AMPI	NA
isolator unit	GRASS	Model SIU5

### Experimental model and study participant details

All experimental procedures were performed under the NIH Guide for the Care and Use of Laboratory Animals and were approved by the Instituto de Neurobiología at Universidad Nacional Autónoma de México (No. 043). C57BL/6 male mice (8–12 weeks old) were used and housed in a temperature-controlled vivarium at the animal facility of the Instituto de Neurobiología, UNAM under a 12/12 h light/dark cycle (lights on at 7 a.m.). For behavioral experiments, mice were water-restricted to 1 mL/day with free access to food.

### Method details

#### Apparatus

The apparatus (18 × 20 × 25 cm) consists of two Plexiglas chambers (A and B) separated by an automatic sliding door. The floor of the A compartment, coupled to a circular vibrator (15 mm), delivered vibrotactile stimulation into the paws. On the wall of the B compartment, there were two nose-poke holes (left and right) connected to infrared sensors that triggered the delivery of water (3 μL) after the mice introduced their noses. The apparatus was controlled with an Arduino microcontroller board and a Visual Basic interface.

#### Categorization task

Mice were trained (100 trials × 16 days) to discriminate vibrotactile frequencies in a two-port choice task following successive approximations using water as a reinforcement. Briefly, mice were located in the A compartment, where they randomly received on the paws vibrotactile stimuli (2 s) of low (20 Hz) and high frequency (80 Hz). Next, the door opened and the mouse was allowed to enter the B chamber to choose the correct port (low frequency, left; high frequency, right) to obtain a reward (water). A correct trial was considered when the mouse chose the correct port. Once the animals reached the criterion of correct trials (80%), we assessed their performance by categorizing new stimuli at varying intermediate test frequencies (24, 30, 36, 44, 54 and 66 Hz) as high or low. These vibrotactile stimuli were selected using a logarithmic spacing between the two category-defining frequencies (20 and 80 Hz) following the Weber-Fechner law.

During the evaluation sessions, a correct trial was considered when the animal selected the left port after receiving test frequencies closer to 20 Hz (20, 24, 30, and 36 Hz), and the right port in response to receiving frequencies closer to 80 Hz (44, 54, 66, and 80 Hz). The correct port choices are presented either as the total number of correct trials or as the probability of choosing the correct port presented in psychometric curves. All the animals were tested across several evaluation sessions under control, CNO, and vehicle conditions, following that order. The Behavioral performance was quantified by plotting the probability of right/left port response (P) as a function of the stimulus frequency and generating a Psychometric Curve Fitting of Individual session data using a standard four-parameter logistic sigmoidal function (Boltzmann fit).

#### Virus injection

The animals were anesthetized with isoflurane (isoflurane/O_2_ gas mixture 5% for induction, 1% for maintenance) and placed in a stereotaxic apparatus, where the temperature of the animal was maintained at 34 °C. The surgery area was cleaned (70% alcohol and betadine) before every procedure, and eye ointment was applied to prevent drying out. Once the animal was stable, a midline incision was made in the head, exposing the skull.

We use stereotaxic coordinates to target S2 (Paxinos G, AP = −1 mm, ML = 4 mm, depth = 1–2 mm) for viral injections of AAV5-gfaABC1D-cyto-GCaMP6f (Addgene, 52925) and pAAV-GFAP-hM3D(Gq)-mCherry (Addgene, 50478). We used a glass graduated pipette (tip diameter 10–25 μm; BlauBrand IntraMARK) connected to a pneumatic pump (PV830, WPI) to deliver 250 nL (flow rate: 20 nL/min). Once the injection was complete, the pipette was left in place for 10 min to prevent leakage of the virus. After the virus was injected, the skin was sutured, and carprofen (2.5 mg/kg) was administered (s.c.) for pain relief before returning the animal to its home cage for recovery.

#### Chemogenetic modulation of S2 astrocytes

We used hM3D(Gq) DREADDs expressed under the GFAP promoter to modulate the calcium activity of S2 astrocytes. To activate these receptors, CNO (APExBIO, cat: B8466, United States) was administered intraperitoneally (0.2 mL) at 1 mg/kg body weight, diluted in saline, a dose that have been previously reported to be effective.^18^^,^^44^ For freely moving animals (electrophysiological recordings and categorization task), CNO was administered 5 min before the start of the evaluation. When the vehicle was administered, the same procedure was done for saline administration instead of CNO.

#### Immunohistochemistry

Animals were euthanized with pentobarbital (PISABENTAL, 45 mg/kg i.p.), perfused with cold phosphate buffer (PBS), and then fixed in 4% paraformaldehyde (PFA) diluted in PBS. The brains were extracted and left in PFA for 12 h to ensure complete tissue fixation. Coronal slices (50 μm thick) were obtained using a cryostat (Leica 3050S). The tissue was permeabilized with 0.5% Triton X-100 (Sigma Aldrich, Cat# X100) and then blocked for 2 h at room temperature in 100 mM PBS containing 4% normal goat serum (Gibco). Primary antibodies were diluted in PBS (Mouse anti-NeuN, 1:1500, Millipore, cat: MAB377, rabbit anti-ALDH1L1 1:500, Abcam, cat: ab177463) containing 1% NGS for 48 h at 4°C. Then the tissue was incubated with secondary antibodies (1:500 goat anti-Rabbit Alexa 488, Thermo fisher scientific #A11008, and 1:500 goat anti-Mouse Alexa 647, Thermo fisher scientific #A21235) for 2–3 h at room temperature in PBS containing 3% NGS and 0.3% Triton X-100. After the incubation, the tissue was washed with PBS and mounted in slides with SlowFade Gold Antifade Mountant (Thermo Fisher Scientific, S36937).

#### Calcium imaging

Astrocytes expressing GCaMP6f were imaged using miniature fluorescence microscopes (Miniscope V4, UCLA). For cellular resolution, a microprism (1.5 mm, Tower Optics) was implanted in S2 under isoflurane (1% isoflurane/O_2_ gas mixture) 3 weeks before calcium imaging. After this period, a field of view (FOV) of 700 × 400 μm was selected with an optimal signal, and the LED intensity and gain were adjusted for each animal to achieve a good signal-to-noise ratio. The baseplate was secured with dental acrylic, allowing stable imaging of the same FOV. For calcium imaging, the animals were anesthetized and placed in a stereotaxic frame for temperature and anesthesia control (isoflurane 0.5%). Calcium imaging was acquired at 10 Hz with a resolution of one pixel per micron. For calcium signal analysis, regions of interest (ROIs) were manually delineated in Fiji using Standard Deviation and Maximum Intensity projections of the full image stack. High-contrast rendering was applied to maximize visualization of fluorescence fluctuations in active astrocytes. ROIs were selected from stacks acquired in both control and CNO sessions, then merged into a unified pool and consistently applied to extract mean fluorescence intensity across all time-series recorded per animal. The fluorescence signals were z-scored relative to the baseline period (mean = 0, SD = 1). Each trial was smoothed with a 2-s moving window and fitted to a Gaussian model with a linear term to estimate time-to-peak, amplitude, and duration. A response was considered evoked by sensory stimulation if the fit of the signal reached an R2 < 0.45, the peak occurred between 3 and 20 s post-stimulus, and the normalized amplitude exceeded 0.5. This approach discriminates stimulus-induced transients from non-evoked baseline oscillations. Only valid trials were used to estimate the response probability and the weighted AUC.

Astrocytes were classified into three groups based on the sensory-evoked calcium responses (AUC) following CNO administration relative to the control condition: astrocytes that were activated by CNO (CNO > Control), inhibited by CNO (CNO < Control), or unaffected by CNO (CNO = Control). For each subpopulation, k-means was used to classify astrocytes based on their response profiles across different test frequencies. The optimal number of clusters for each subpopulation was determined using the Calinski-Harabasz index, which maximizes inter-cluster variance and minimizes intra-cluster variance.

#### Optodrive stereotaxic implantation

Mice were anesthetized with isoflurane (1% for maintenance and 3% for induction) and secured in a stereotaxic frame (Stoelting). Ophthalmic ointment (hydrocortisone, neomycin, and polymyxin B) was applied to prevent corneal drying. Lidocaine (0.03 mL, subcutaneous) was administered at the incision site for local analgesia, and enrofloxacin (0.4 mL/kg) was injected for three days postoperatively as antibiotic prophylaxis. OptoDrive electrodes were coated with CellTracker CM-Dil (C7001, Invitrogen) and implanted into the S2 cortex (AP = -1 mm, ML = 3.7 mm, DV = -1-2 mm). The implant was fixed to the skull using an initial layer of adhesive cement (C&B Metabond; Parkell), followed by cyanoacrylate glue (Loctite) and dental acrylic (Nic Tone, MDC Dental).

#### Freely moving electrophysiological recordings

*In vivo* multichannel electrophysiological recordings were performed in freely moving mice using an optodrive array, the array comprises an array of 16 tungsten wires coated (35 μm diameter).^45^ Extracellular neural signals were acquired using a 64-channel PZ5 Neurodigitizer amplifier (Tucker-Davis Technologies, FL, USA) connected to an RZ2 BioAmp Processor (Tucker-Davis Technologies, FL, USA). Data were digitized at 50 kHz with 28-bit resolution and band-pass filtered between 0.5 and 8 kHz. Spikes were detected online using voltage–time threshold windows. Units were considered single neurons when spike amplitudes exceeded 50 μV and were 3.5–4 standard deviations above the mean channel signal. Detected spikes were exported with OpenBridge software (Tucker-Davis Technologies, FL, USA) and subsequently sorted offline using Offline Sorter (Plexon, TX, USA). Only timestamps from the offline-sorted units were used for further analyses.

For the recordings, the animals were placed in the behavioral apparatus where they received vibrotactile stimulation on the paws. Neuronal activity was recorded for around 80 min. Briefly, saline was administered (i.p.) following a 10-min baseline period. After 15 min, 60 trials of vibrotactile stimulation were delivered over 10 min. Each trial consisted of 2 s of vibrotactile stimuli separated by an inter-trial interval of 8 s (10 s total). The frequency of the vibrotactile stimuli was randomly selected from 20, 36, 44, or 80 Hz, with 15 trials per frequency. All animals were evaluated under both saline and CNO conditions.

#### Quantification of sensory adaptation

For each S2 neuron (*n* = 106), peri-stimulus time histograms (PSTHs) were constructed by aligning spike times to the onset of each vibrotactile stimulus and binning firing rate in 10-ms bins over a window from −0.5 to 3 s relative to stimulus onset (350 bins per epoch). Separate PSTHs were computed for each of the four stimulation frequencies (20, 36, 44, and 80 Hz) and for each condition (control/saline and CNO). Slopes were computed directly on the trial-averaged firing-rate PSTHs, without additional smoothing.

Sensory adaptation was quantified as the slope of the firing rate during the 2-s stimulation window. For every neuron, frequency, and condition, an ordinary least-squares line was fit (using polyfit MATLAB function) to the firing rate over the 0–2 s interval (200 bins), and its slope (in Hz s^−1^) was taken as the adaptation index, with negative values indicating a progressive decrease in firing rate (adaptation) and positive values an increase. The standard error of each slope was obtained from the regression residuals as SE = √[(SSE/(N−2))/S_xx_], where SSE is the residual sum of squares, N the number of bins in the fit window, and S_xx_ the sum of squared deviations of the time axis. To classify the effect of CNO on a per-neuron basis without an arbitrary cut-off, the CNO slope was compared with the saline slope for each neuron and frequency using a two-sided z-test based on the pooled standard error of the two independent regression slopes:

z = (m_CNO − m_saline) / √(SE^2^_saline +SE^2^_CNO)

The resulting *p*-values were corrected for multiple comparisons across all neuron × frequency tests using the Benjamini–Hochberg false-discovery-rate procedure (FDR, α = 0.05). Each neuron was then labeled, for each frequency, as showing a significantly *decreased* slope (more negative under CNO), a significantly *increased* slope (more positive under CNO), or *no change* (difference not significant after FDR correction). At the population level, slopes were compared between conditions for each frequency with Wilcoxon signed-rank tests, and results are reported as medians. All analyses were performed in MATLAB (R2025b) using custom routines.

#### Electrophysiological recordings in anesthetized animals

Male mice (3 months, 26–30 g) were anesthetized with a mixture of isoflurane/O_2_ (1%), placed in a stereotaxic frame, and maintained at a constant temperature (37°C) with a thermic pad. During the experiment, the depth of anesthesia was assessed using the tail and toe pinch reflexes. Neuronal recordings were performed in S2 (AP: −1 mm, ML: 4 mm relative to bregma; depth: 1.5 mm). For this, a cranial window was performed, and *in vivo* extracellular recordings (multiunitary) were done with a 32-channel silicon-based multielectrode probe (A4x8-5mm-50–200–177-A32 configuration; Neuronexus Technologies) positioned in place with a micromanipulator (Luigy & Neumann, Mini Compact Unit). Sensory-evoked responses were induced by electric stimulation (45 stimuli) of the receptive field applied by two fine needles inserted (subdermally) into the contralateral forepaw of the cranial window. The stimulus consisted of three pulses (P1, P2, and P3) delivered at 5 Hz (5V, 1 ms) using a stimulator (Master 8, AMPI) connected to an isolator unit (GRASS SIU5). The electrophysiological signals were amplified and band-pass filtered (300 Hz-5 kHz) to isolate the unitary activity. We used OpenEphys software to digitize the signal at 20 kHz and to sort based on waveform characteristics and principal component analysis. Peristimulus time histograms (PSTH) were performed for the sensory-evoked responses, and the resultant fire probability (p) was analyzed at a temporal resolution of 5 ms per bin.

For electrophysiological analysis, the spontaneous and sensory-evoked activity were computed for each neuron. Spontaneous activity was defined as the neuronal activity that occurred within 200 ms before the delivery of the stimuli. The sensory-evoked responses were quantified as the activity recorded within 50 ms post stimulus. Only neurons with an increase in sensory-evoked calcium responses due to CNO administration were included in the analysis. For the calculation of the signal-to-noise we compute the normalized mean of the evoked response in relation to the spontaneous activity. For sensory adaptation, the normalized mean of the evoked response was expressed relative to the first stimulus of each pulse and a linear regression was fitted to the data to calculate the X-intercept for each condition.

### Quantification and statistical analysis

Normality of the data was evaluated using the Shapiro–Wilk test prior to statistical comparisons. For within-animal longitudinal contrasts between baseline and CNO conditions, paired Student’s t-tests were applied to two-group data; unpaired Student’s t-tests were used for between-group contrasts. For multi-frequency behavioral sessions, one-way repeated-measures ANOVA followed by Tukey’s post-hoc test was applied for pairwise comparisons across test frequencies. When normality assumptions were violated, paired comparisons were analyzed using Wilcoxon signed-rank tests, and unpaired comparisons were analyzed using Mann–Whitney U tests. Two-tailed tests were used throughout for statistical significance. Asterisks in the figures denote ∗*p* ≤ 0.05. Data are presented as mean ± SEM, and the exact value of n and what it represents are reported in each figure legend. Astrocyte subpopulations were classified using k-means clustering; the optimal number of clusters per subpopulation was determined using the Calinski–Harabasz index, which maximizes inter-cluster variance while minimizing intra-cluster variance. Statistical analyses were performed with custom-written routines in MATLAB (version R2025b, MathWorks, https://www.mathworks.com/products/matlab.html).
