# Supplementary material for: Astrocytes improve tactile categorization by modulating sensory processing in neurons
Source: iScience. 2026 Oct 29;29(11):117972. doi: 10.1016/j.isci.2026.117972 (PMC13738171; doi:10.1016/j.isci.2026.117972)
Supplement: Supplementary file 1 [file mmc1.pdf]

iScience, Volume *29*

Supplemental information

Astrocytes improve tactile categorization

by modulating sensory processing in neurons

Alejandra Calero-Vargas, Frida Higinio-Rodríguez, Rafael Olivares-Moreno, Luis Alfredo Rodríguez-Blanco, Victor de Lafuente, Ranier Gutierrez, Gerardo Rojas-Piloni, Oswaldo Pérez, and Mónica López-Hidalgo

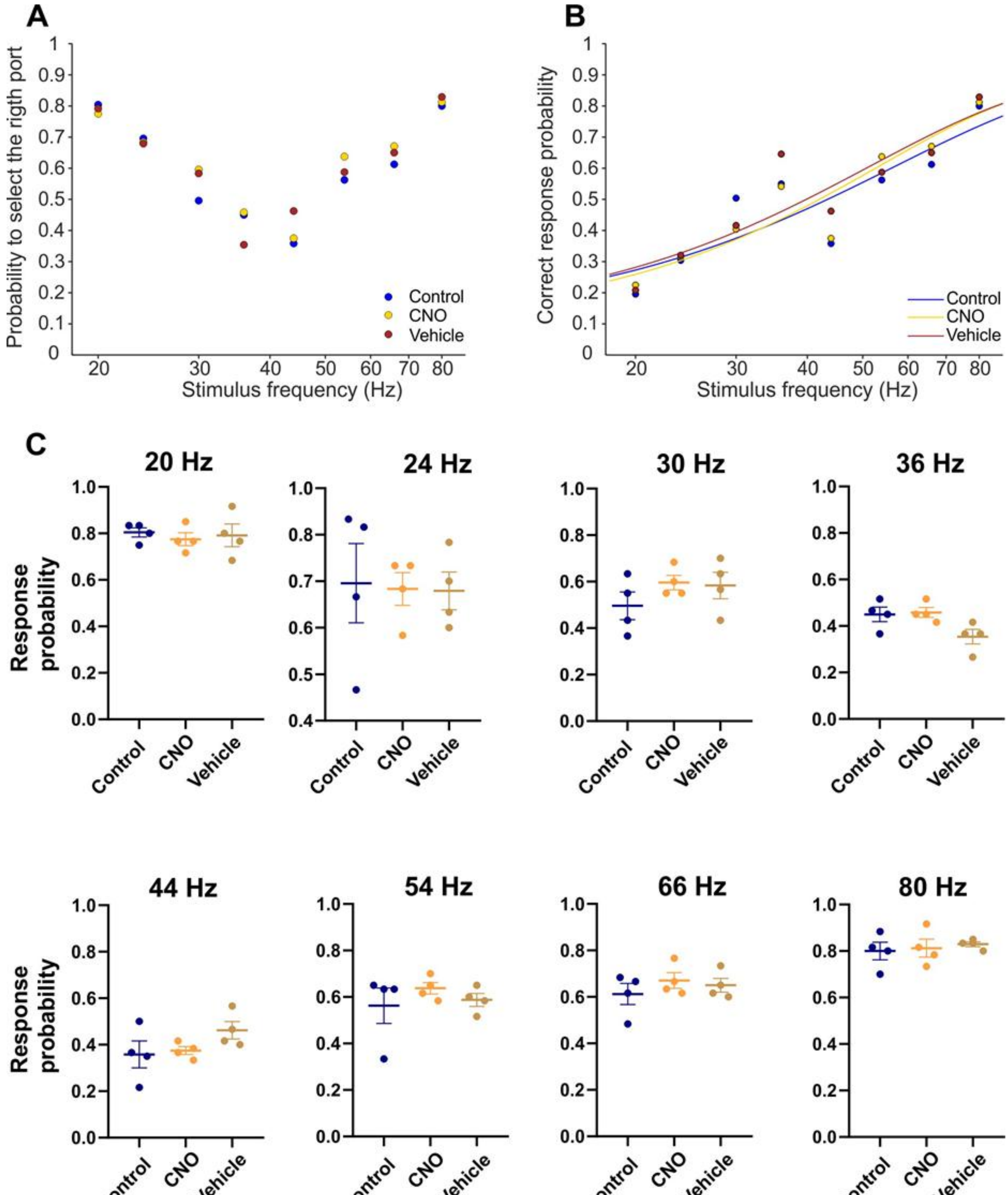

Figure S1. Chemogenetic modulation of V1 cortical astrocytes does not change mice performance in a vibrotactile categorization task. To determine whether the behavioral effects of chemogenetic modulation of S2 astrocytes generalize to other sensory cortical regions, we performed the same vibrotactile frequency discrimination task (see Methods: Vibrotactile categorization task) in mice expressing DREADDs (hM3D(Gq)) under the GFAP promoter in V1 (primary visual cortex) astrocytes. **A,** probability of correct port selection as a function of stimulus frequency (20, 24, 30, 36, 44, 54, 66, 80 Hz), under control (blue), CNO (orange), and vehicle (brown) conditions **B,** Psychometric curves fit showing the probability of correct port selection as a function of stimulus frequency under the three conditions. Curves overlap closely, indicating no significant shift in the perceptual discrimination threshold after chemogenetic activation of V1 astrocytes. **C,** Comparison of the response probability of correct port selection of the eight stimulus frequencies throughout the session in control, CNO, and vehicle conditions. No significant differences were found in the three groups (n=4; one-way repeated-measures ANOVA, $^{*}p \leq 0.05$).

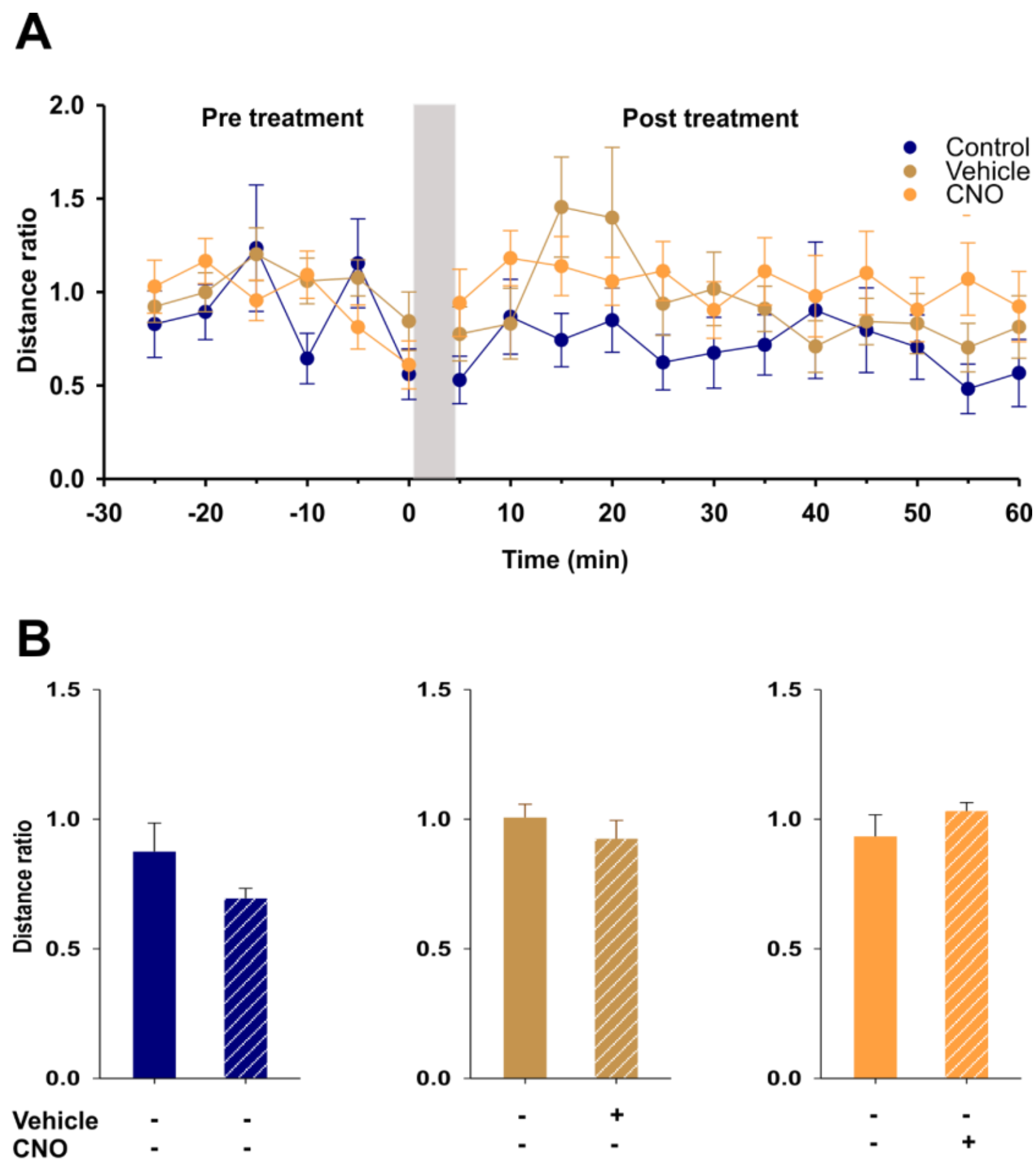

Figure S2, CNO administration does not produce off-target effects on voluntary locomotor activity. Three-month-old male wild-type C57BL/6J mice (n = 5 per group) were housed individually in cages equipped with a freely rotating running wheel (LE905; Panlab, Harvard Apparatus; wheel diameter 16 cm; revolutions monitored continuously via dedicated acquisition software and binned every 5 min). After at least 5 days of acclimation to the vivarium and 1 day of habituation to the wheel cages, baseline wheel running was recorded for 30 min (pre-treatment, "pre-CNO" window) immediately followed by intraperitoneal injection of CNO (1 mg/kg in 0.2 mL sterile saline; yellow/orange trace), saline (0.2 mL; brown trace), or handling only (no injection; blue trace). Post-treatment wheel running was recorded for 60 min (post-CNO window) and testing was repeated on three consecutive days. **A,** Time course of the normalized distance ratio across the full 90-min recording session in Control (no injection/handling only; blue), Vehicle (intraperitoneal saline injection; brown), and CNO (CNO i.p. injection; yellow) groups. The shaded gray region marks the 5-min injection/handling window (t = 0). **B,** Group comparisons of mean distance ratio between the pre and treatments windows. Students t-tests show no significant differences due to CNO injection/handling in any of the three groups (*p > 0.05), confirming that the systemic CNO dose used in our DREADD experiments does not induce nonspecific locomotor or motivational effects. Data are presented as mean ± SEM; n=5.

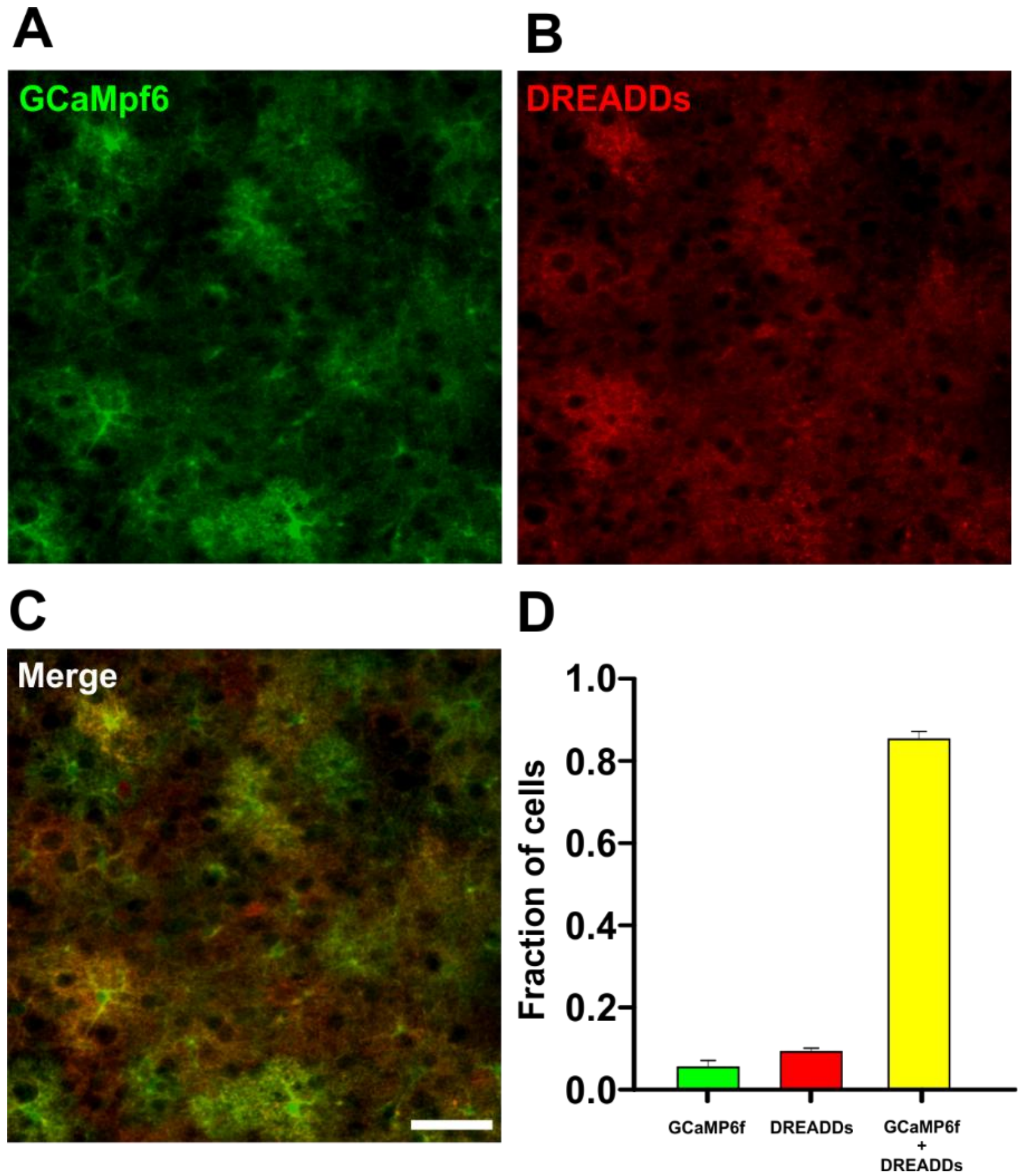

Figure S3. Specificity of viral expression in S2 cortical astrocytes. **A-C**, Representative micrographs of S2 cortical astrocytes co-transduced with AAV-GCaMP6f (green) and AAV-hM3Dq-mCherry DREADDs (red), **A**. Immunofluorescence against GFP identifying GCaMP6f expressing cells (chicken anti-GFP 1:400, Invitrogen A10262). **B**. Immunofluorescence against mCherry labelling DREADDs expressing cells (rabbit anti-mCherry 1:400, Invitrogen PA5-34974). **C**. Merged image of A and B showing extensive colocalization of both markers. Brains were PFA-perfused, sectioned at 50 µm, and imaged by confocal microscopy (see Methods). Scale bar = 50 µm. **D.** Quantification of co-transduction efficiency across n = 9 fields from n = 3 animals shows ~5% GCaMP6f-only (green), ~10% DREADD-only (red), and ~85% double-positive (yellow), confirming dual-transduction of individual astrocytes. Bars = mean ± SEM.

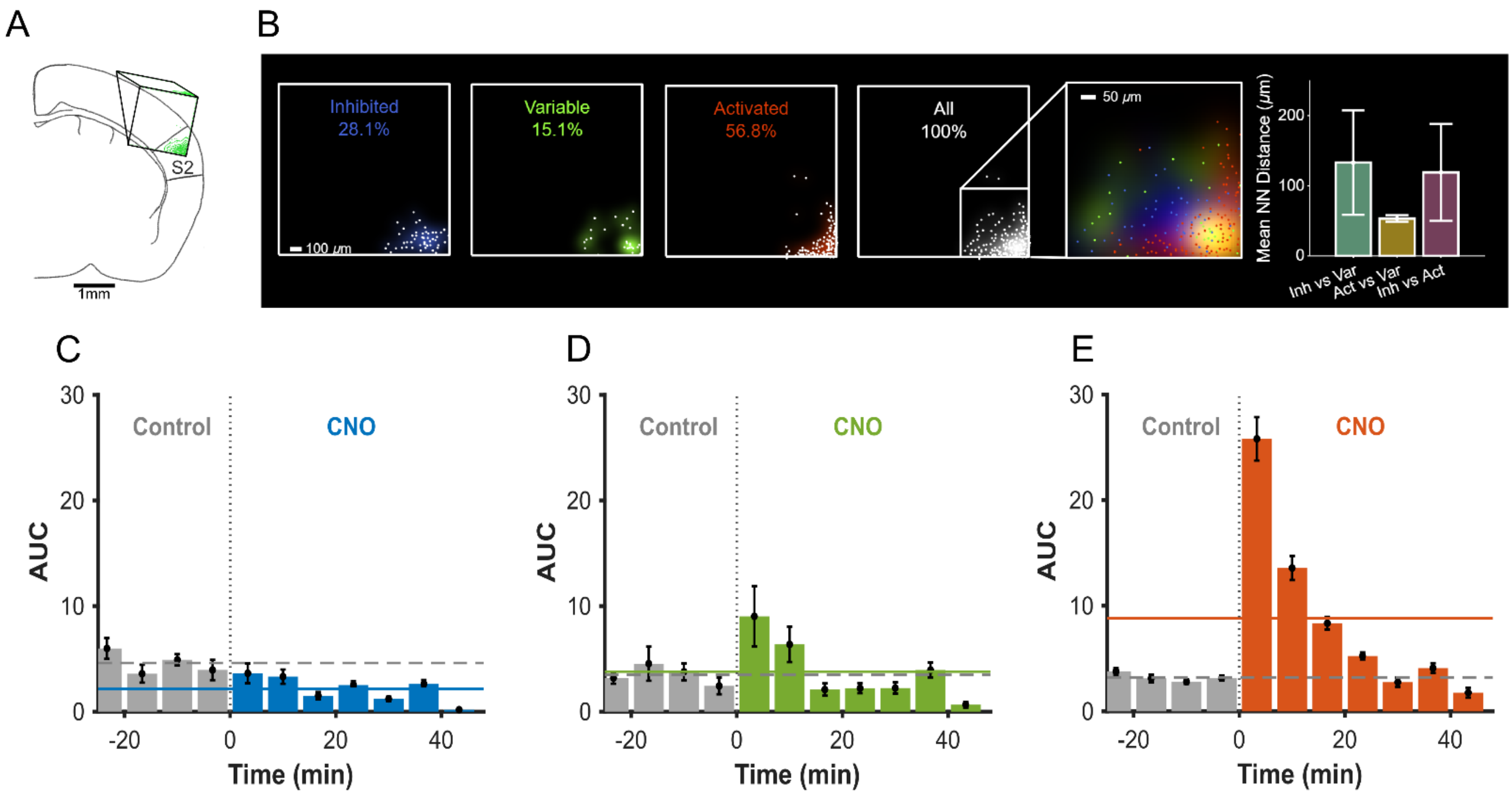

Figure S4. Spatial distribution and temporal dynamics of astrocyte subpopulations in S2 during DREADDs activation. A, Schematic representation of a coronal section of the right hemisphere, indicating the estimated location of the microprism in the secondary somatosensory cortex. The green shaded area delineates the field of view used for calcium recording acquisition in anesthetized subjects. Scale bar: 1 mm. n = 4 mice. **B**, Spatial distribution of astrocyte centroids, classified according to their global calcium response to CNO administration: Inhibited, Variable, and Activated. The fourth panel shows the cumulative distribution of all analyzed astrocytes. The 100µm scale bar applies to the first four panels. The inset illustrates the spatial overlap of the three subpopulations, complemented by a bar graph detailing the mean distance to the nearest neighbor between pairs of groups. Data are presented as mean ± SEM. Temporal dynamics of the Area Under the Curve (AUC) of calcium activity evoked by vibrotactile stimulation of Inhibited Astrocytes (**C**), Variable Astrocytes (**D**), and Activated Astrocytes (**E**). The horizontal axis represents experimental progression in blocks of 10 trials in control and after CNO administration. The dashed gray lines indicate the global mean AUC during control; the solid colored lines indicate the mean AUC under CNO for each subpopulation. Data are expressed as mean ± SEM. n = 171 astrocytes from 3 mice.

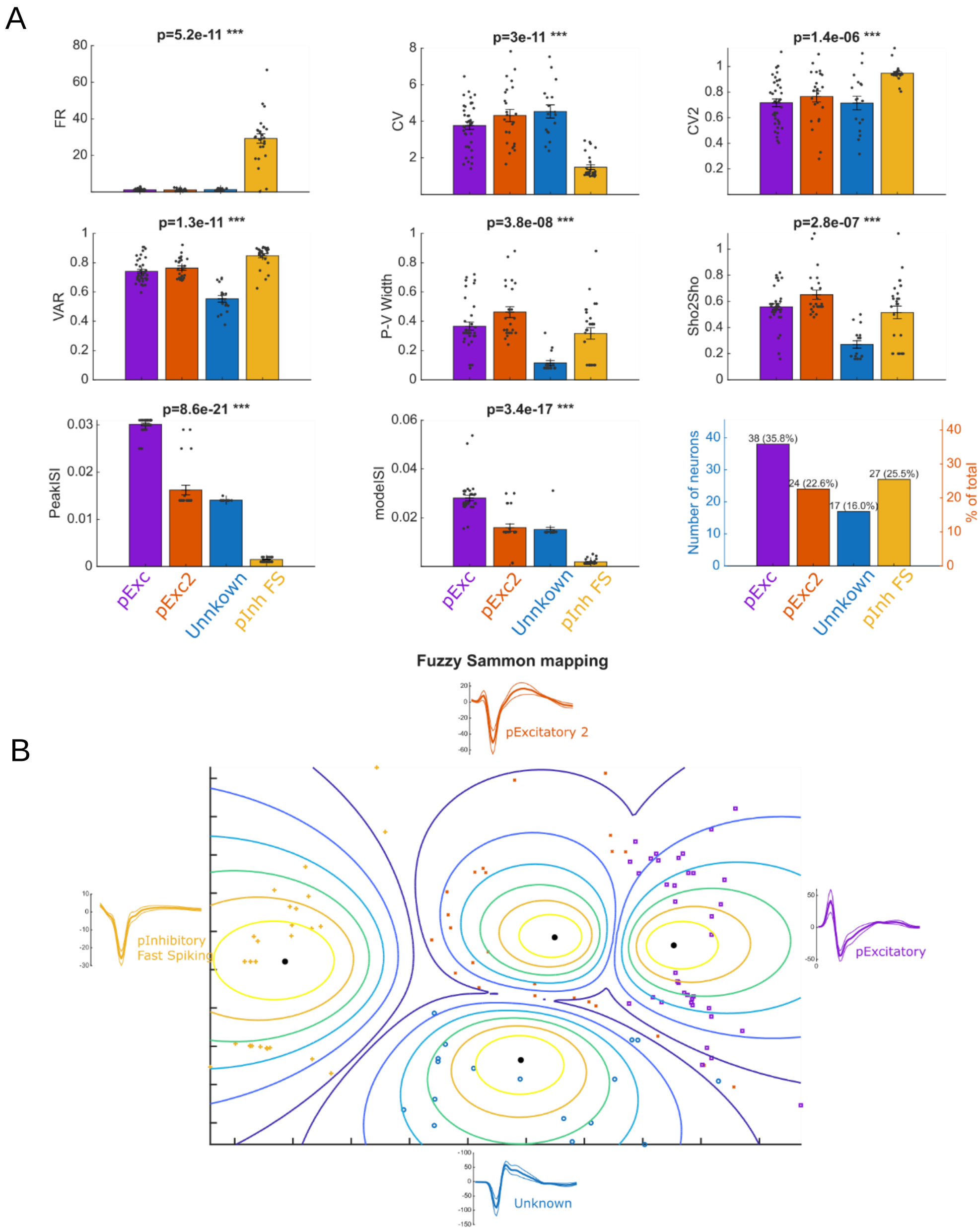

Figure S5. Single units were classified into putative cell types using fuzzy Sammon clustering-based waveform analysis. A, The analysis identified fast-spiking inhibitory neurons (pInh FS), two groups with classic excitatory features (pExc and pExc2; low firing rates and a delayed peak inter-spike interval), and a fourth group with low firing rates but narrow peak-to-valley widths. Because this last group could correspond to either non-fast-spiking inhibitory inter-neurons or excitatory cells, it was labeled “Unknown”. Neurons were classified according to eight features: firing rate, coefficient of variation (CV), coefficient of variation 2 (CV2) of the inter-spike-interval (ISI), valley-to-amplitude ratio (VAR), valley-to-peak width (V-Pwidth), shoulder to shoulder, the peak of the ISI distribution and its mode. Firing rate was calculated as the number of recorded spikes divided by session duration. CV2 was calculated for each adjacent ISIs pair, and the average CV2_ISI for the entire session was used. VAR was calculated as the absolute value of the first valley in the waveform divided by the difference between its minimum value and the subsequent peak. For each neuron recorded (m= rows), these eight features (n=columns) were computed resulting in an m x n data matrix. Values in each column of the _data_matrix were normalized to (0,1) by using the following formula [n - min(n)]/[max(n) - min(n)], classified into groups with similar attributes by using the fuzzy cluster algorithm and visualized by using PCA as seed followed by the fuzzy Sammon’s mapping plot as described in the Fuzzy Clustering and Data Analysis Toolbox. For more details, see (Tellez et al., 2012). **B**, For each cell type, the larger the ellipsoid, the smaller is the membership’s certainty. Insets display mean waveforms and SE for each putative cell type.
